# Supplementary material for: An on-chip phased array for non-classical light
Source: Nat Commun. 2025 Jul 29;16:6849. doi: 10.1038/s41467-025-61886-9 (PMC12307807; doi:10.1038/s41467-025-61886-9)
Supplement: Supplementary file 1 — Supplementary Information [file 41467_2025_61886_MOESM1_ESM.pdf]

Supplementary information for "An on-chip phased array for non-classical light"

Volkan Gurses,[1,2,*] Samantha I. Davis,[2,3] Raju Valivarthi,[2,3]
Neil Sinclair,[3,4] Maria Spiropulu,[2,3] and Ali Hajimiri[1]

[1] *Division of Engineering and Applied Science, California Institute of Technology, Pasadena, CA, USA*
[2] *Division of Physics, Mathematics and Astronomy, California Institute of Technology, Pasadena, CA, USA*
[3] *Alliance for Quantum Technologies (AQT), California Institute of Technology, Pasadena, CA, USA*
[4] *John A. Paulson School of Engineering and Applied Sciences, Harvard University, Cambridge, MA, USA*

CONTENTS

I. PIC components 1
A. Metamaterial antenna 1
1. Near-field and far-field characterization 2
2. Loss characterization 3
B. Quantum coherent receiver 6
1. QRX design 7
2. Insertion loss characterization 8
3. Common-mode rejection ratio characterization 8
4. High shot noise clearance configuration 9
5. High bandwidth configuration 10
C. Thermo-optic phase shifter 11

II. PIC system characterization 11
A. 32-channel common-mode rejection ratio (CMRR) 12
B. 32-channel shot noise clearance (SNC) 12

III. On-chip squeezing analysis 12

IV. Squeezed light source characterization 13

V. High bandwidth measurement of squeezed light 17

VI. Phase calibration algorithm 17

VII. Measurement characterization 18
A. Squeezed light imaging 18
1. Data acquisition and analysis 18
2. Squeezing parameter estimation 19
3. Phase estimation 19
4. Channel effective efficiency estimation 19
5. Classical imaging 20
B. Beamforming and pump power sweep 21
1. Classical channel sweep 21
C. Beamwidth 21
1. Classical beamwidth 22
D. Field of view 22
1. Classical field of view 23

VIII. Loss budget and improvements 23
A. System loss budget 23
1. Source loss 23
2. Free-space loss 23
3. On-chip loss 23
4. RF loss 24
B. Measurement loss budget 24
1. Squeezed light imaging 24
2. Wavefunction engineering 24
C. Loss improvements 24

IX. Scaling analysis and applications 25
A. Loss analysis 26
B. Quantum-enhanced sensing 26
C. Quantum key distribution 27

References 29

* Corresponding author: gurses@caltech.edu

I. PIC COMPONENTS

Our photonic integrated circuit (PIC) is one of the largest-scale PICs with more than 1,000 functional components, as shown in Fig. S1. Photos of the chip on top of a penny are shown in Fig. S1a, and the packaged photonic-electronic system is shown in Fig. S1b. Here, we provide a description of the PIC components and their characterization.

A. Metamaterial antenna

The metamaterial antenna (MMA) uses two etch layers in Si to implement the sub-wavelength gratings: 220 nm thick layer for the waveguide and 90 nm thick layer for the grating teeth. The scattering strengths of the sub-wavelength gratings in the antenna were apodized to mode match the amplitude profile of an incident beam as much as possible. There are multiple options (apodizing grating duty cycle, width, etc.) to tune the scattering strength of the gratings. Each option has a lower and upper bound on the scattering strength per area in the antenna. Accessing the greatest range between the upper and lower bounds for the scattering strength per area requires using multiple options at once. Therefore, three regions with different options were employed.

The first region ($x = 47$ μm to $x = 347$ μm) adjusts the duty cycle of the grating. The second region

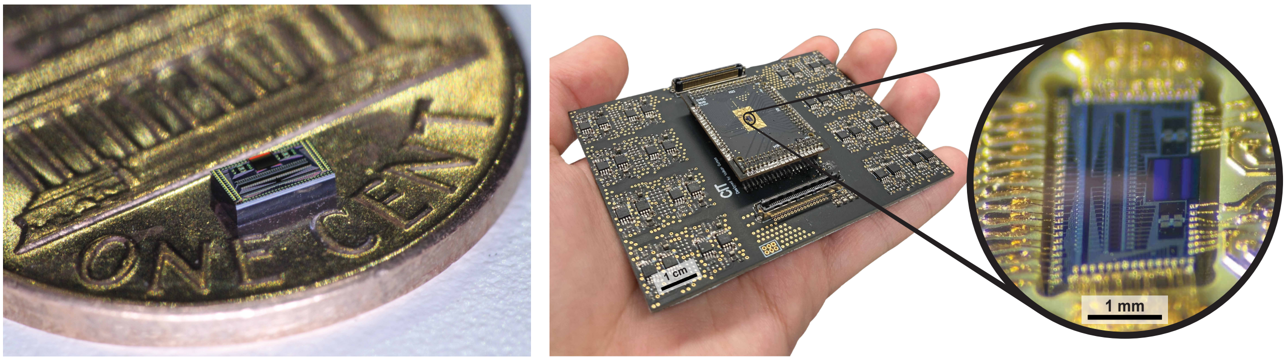

SUPPLEMENTARY FIG. S1. Photo of the PIC on top of a penny (left). Photos of the packaged photonic-electronic system comprising the chip wirebonded to an interposer interfaced with an RF motherboard (right). The system can be packaged as a handheld device enabled by the integration and packaging of photonics and electronics.

($x = 347$ μm to $x = 547$ μm) adjusts the width of the grating. The third region ($x = 547$ μm to $x = 597$ μm) expands the grating width to span the entire antenna. Since the effective index of the grating is different than the effective index of the waveguide, adjusting the duty cycle and width also requires adjusting the period to have 2π phase propagation in one grating period. The scattering strength and the grating period for coupling at 2° center angle as a function of the grating duty cycle and width are shown in Fig. S3.

1. *Near-field and far-field characterization*

Two different setups are used to characterize the near-field and far-field pattern of the MMA. For both setups, a single MMA test structure is used. This test structure is a standalone MMA directly connected to a photodiode for optical in, electrical out measurement. Light coupled into the MMA is routed to a photodiode away from the antenna to prevent optical crosstalk. The MMA is illuminated with a source, and the photocurrent from the photodiode is measured to probe the coupled power. For the near-field measurement, a lensed fiber (Oz Optics TPMJ-X-1550-8/125-0.4-10-1-12-1) with 1 μm spot size and 12 μm working distance is placed on a positioner and is aligned in the vertical direction (z) to focus light onto the antenna at a certain illumination angle. The lensed fiber is then swept in horizontal directions (x and y) to scan the physical footprint of the antenna. For every position, the photocurrent is recorded. This measurement is then compared with the near-field pattern obtained from the FDTD simulation. Since the mesh size of the FDTD simulation is smaller than the spot size of the lensed fiber, the simulated pattern is convolved with the point spread function of the 1 μm spot size lensed fiber. The measured and simulated MMA near-field patterns are shown in Fig. S4a. The primary discrepancy between measurement and simulation is the dip in scattering observed in the middle part of the antenna ($x \simeq 200$ μm to $x \simeq 350$ μm). To rule out the interference effects in the measurement as the cause of this dip, the near-field characterization measurement was done at a range of wavelengths (1500 nm to 1600 nm) and at a range of illumination angles (-45° to 45°), but the dip was observed in the same location for all measurements. The dip could have been caused by faulty fabrication of the gratings due to the limited lithographic resolution, such as image errors causing the rectangular gratings to round off or variations in the grating sidewall slope and layer thickness, leading to a different scattering strength than the one in the simulation. These errors can be accounted for in the simulation and layout (e.g. optical proximity correction) in future designs to make the design robust against fabrication errors. Despite the discrepancy, the MMA scatters light over a sufficiently large area for low-loss coupling with a 200 μm beam diameter collimator (Oz Optics LPC-07-1550-8/125-P-0.2-1.01CL-60-3A-1-1).

For the far-field characterization, the chip was placed on an auto-alignment stage with its XY plane parallel to the XY plane of the positioner holding the collimator. A collimator is used to illuminate the antenna with a 200 μm collimated beam diameter. The collimator is centered on the antenna so that the modal overlap between the incident beam and the antenna is maximized, and the angle of the collimator is scanned in two orthogonal directions (θ_x and θ_y) to measure the orthogonal radiation patterns. The far-field pattern was also simulated with the FDTD simulation assuming an ideal planar phasefront illuminating the MMA. This raw simulation data is then convolved with the point spread function of the 200 μm beam diameter collimator to compare with the measurement data. The raw simulated MMA far-field pattern is shown in Fig. 3a and also in more detail in S5. For comparison, the measured and simulated MMA far-field patterns are shown in Fig. S6. The center angle in θ_x, θ_y in simulation is $\theta_x = 1.92°$ and $\theta_y = 0°$. The measurement data is aligned with this center angle. In the comparison, the far-field pattern has a measured (simulated) θ_x of 0.518° (0.639°) and θ_y of 2.79° (3.05°). Despite the discrepancy in the near-

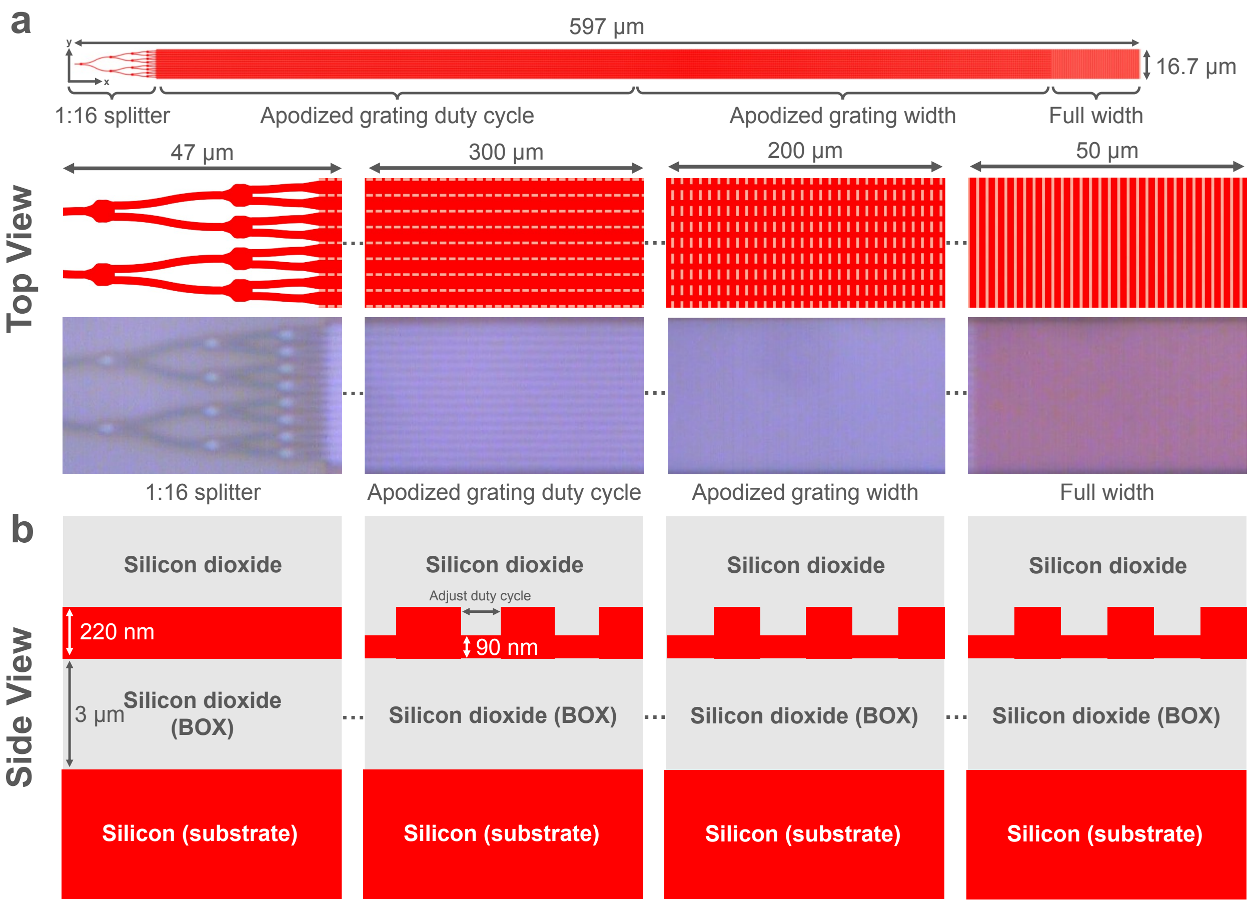

SUPPLEMENTARY FIG. S2. a) Top view rendered drawings and micrographs of the metamaterial antenna showing XY dimensions of the regions. b) Side view rendered drawings of the metamaterial antenna showing X dimensions and the layer stack-up.

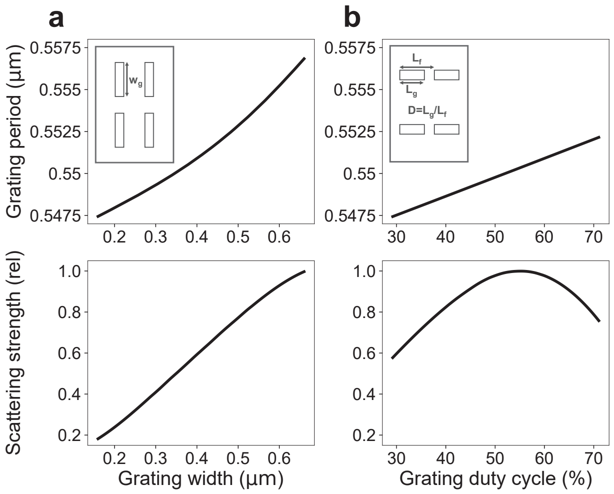

SUPPLEMENTARY FIG. S3. a) Grating period (top) and scattering strength per area (bottom) to couple at 2° for varying grating width. b) Grating period (top) and scattering strength per area (bottom) to couple at 2° for varying grating duty cycle.

field pattern, since the discrepancy happens after the scattering gets sufficiently low, the far-field patterns match relatively well between measurement and simulation enabling low-loss free-space-to-chip coupling.

2. *Loss characterization*

The total loss of the MMA can be split into two categories. The first one is the geometric loss due to the mode mismatch between the free-space mode incident on the MMA and the MMA aperture scattering profile. The second one is the insertion loss characterizing the loss from the front-to-back ratio (the scattering ratio between upward scattering and downward scattering) and the propagation loss through the waveguides and splitters.

a. Geometric loss Using the measured and simulated near-field patterns, geometric loss of the MMA can be characterized for any incident mode. The geometric loss or efficiency (η_g) calculations can be done by using the following modal overlap equation.

$$\eta_g = \frac{\left|\int E_1^* E_2 \, \mathrm{d}A\right|^2}{\int |E_1|^2 \mathrm{d}A \, \int |E_2|^2 \mathrm{d}A} \tag{S1}$$

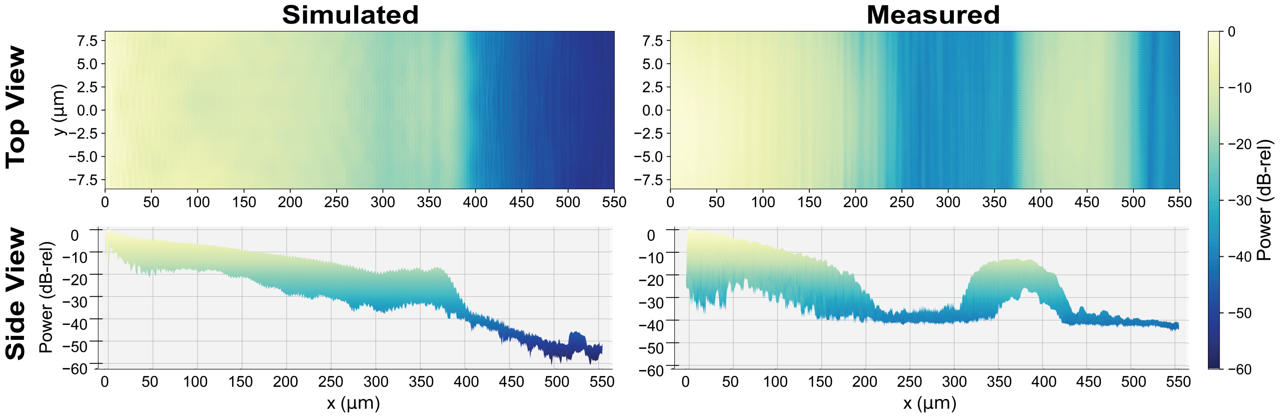

SUPPLEMENTARY FIG. S4. Simulated (left) and measured (right) near-field patterns of the antenna from top view (top) and side view (bottom). The antenna dimensions are the same as the plots.

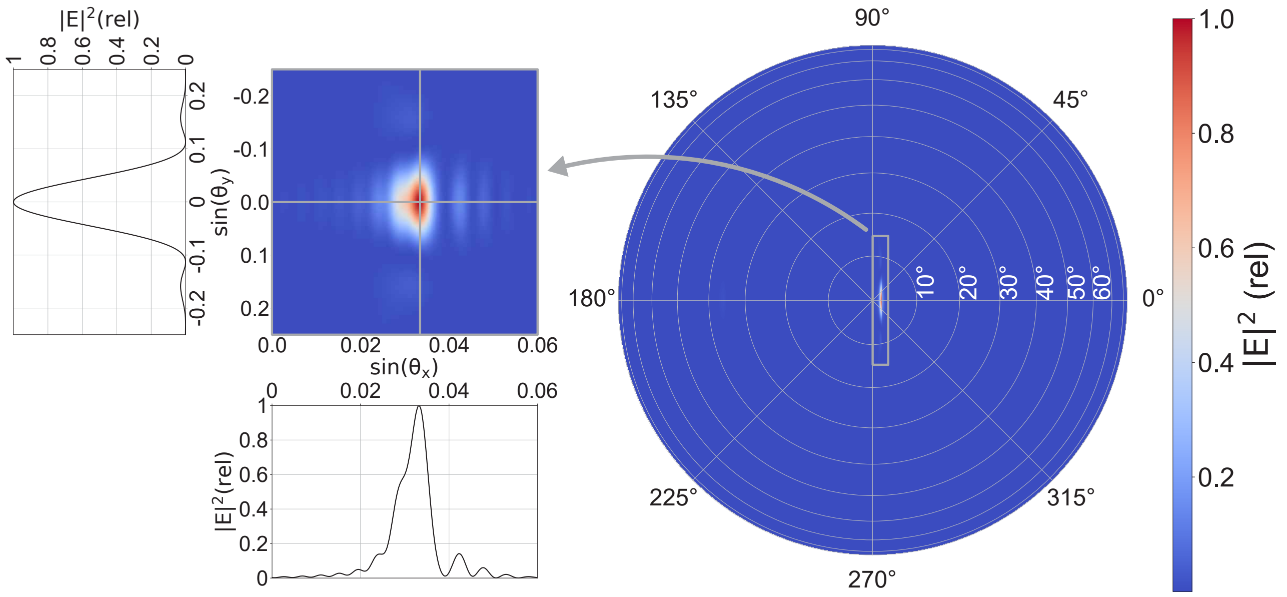

SUPPLEMENTARY FIG. S5. Simulated far-field radiation pattern of the antenna showing diffraction-limited beamwidth with no higher order diffraction (right) and zoomed-in pattern with slices along θ_x and θ_y (left).

where E_1 and E_2 are the electric field profile of the incident mode and the electric field scattering profile of the MMA. Since the radiation pattern is diffraction-limited without any grating lobes, we assume a flat phase front for the antenna scattering profile. With this assumption, Eq. S1 can be rewritten in terms of the measured or simulated near-field intensity pattern as

$$\eta_g = \frac{\int \sqrt{P_1 P_2}\, \mathrm{d}A}{\int P_1 \mathrm{d}A \int P_2 \mathrm{d}A} \tag{S2}$$

where P_1 and P_2 are the intensity profile of the incident mode and the intensity profile of the MMA acquired from simulation or measurement. The intensity profile of a single MMA is arrayed to form the aperture. Intensity profiles of Gaussian beams with varying diameters are also constructed. Then, Eq. S2 is used to calculate the geometric loss. Since each channel can be controlled independently, the profile of the aperture can be tuned by weighing the profile of each MMA. We call this the weighted aperture profile. An example weighted aperture profile for 32 channels optimized for 200 μm beam diameter collimated beam is shown in Fig. S7 along with the intensity profile of the beam. When the weights are not used, namely when each MMA profile has the same amplitude, an unweighted aperture profile is constructed. The unweighted aperture profile for 32 channels is shown in Fig. S7. Across various beam diameters, the minimum geometric loss for the weighted 32 channel profile is 1.01 dB (1.30 dB) for measured (simulated) aperture profile with 150 μm (230 μm) beam diameter. Relevant for the experiments in which unweighted 8 and 32 channels are used, the geometric loss can be extracted. For an optimally aligned 200 μm beam diameter Gaussian beam, the geometric losses for unweighted 8 and 32 channels are 2.18 dB (2.03 dB) and 4.85 dB (4.50 dB), respectively. The geometric loss for

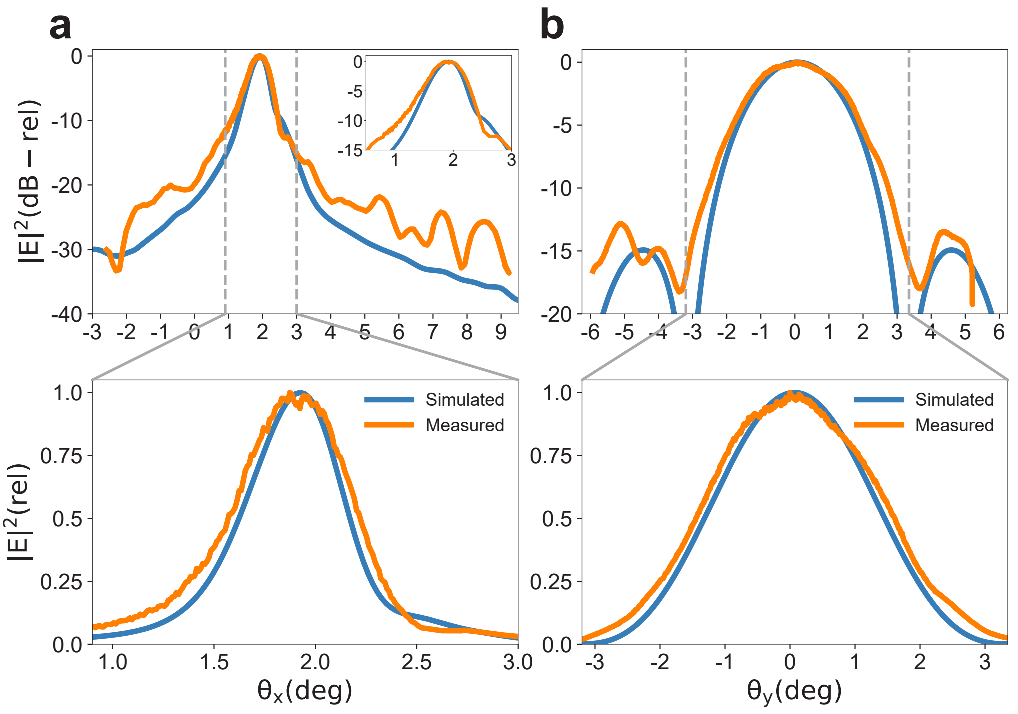

SUPPLEMENTARY FIG. S6. Simulated and measured far-field radiation patterns sliced at the center of the beam along a) θ_x and b) θ_y in dB (top) and linear (bottom) scale. A zoomed-in version of the peak in θ_x is also plotted in the inset of (a). The measured (simulated) 3-dB beamwidths of the antenna along θ_x and θ_y are 0.518° (0.639°) and 2.79° (3.05°), respectively.

weighted 32 channels is 1.14 dB (1.35 dB) for measured (simulated) aperture profiles. This is a greater than 10 dB improvement over previous aperture designs for free-space-to-chip coupling. The discrepancy between simulation and measurement is caused by the aforementioned dip in the measured near-field pattern, making the mode mismatch for the measured antenna lower since it has an active area closer to the area of the 200 μm beam diameter Gaussian beam. The geometric efficiencies characterizing the geometric loss for various apertures across various beam diameters are shown in Fig. S8. These geometric losses can further be reduced with better MMA designs that mode match precisely to an incoming field mode.

As a final point, the weighted 32-channel geometric loss is the loss observed when only the received signal amplitude is considered, without considering the complete signal-to-noise ratio (SNR). Since SNR is proportional to the effective efficiency of squeezed light measurements (see Section VII), the weighted 32-channel loss always characterizes the power loss of classical signals, even when the channels are combined without weights (since sending no input signal to an antenna with non-zero weight doesn't contribute signal to combined signal amplitude but contributes noise to combined noise amplitude). This is the case for the chip loss characterization in Methods and in the next section, where the weighted 32-channel loss is used to de-embed the total loss to extract the antenna insertion loss.

b. Insertion loss Insertion loss of the MMA includes other losses that are not included in the geometric efficiency, such as the loss from downward scattering (scattering light below the antenna) and waveguide propagation losses in the antenna active area and splitters. Insertion loss was characterized by sending 200 μm collimated beam to the chip aperture after setting all QRXs to the unbalanced (100:0) configuration and summing all QRX currents. For 0.452 mW input power, the output current is 0.0615 μA, resulting in a measured loss of 8.66 dB. This loss includes collimator insertion loss, connector loss, QRX insertion loss, waveguide propagation loss, aperture geometric loss, and antenna insertion loss. The collimator insertion loss is 0.8 dB and the connector loss is expected to be <1 dB. QRX insertion loss experimentally measured with a QRX test structure (see Sec. I B) is 1.58 dB. Since the chip loss measurement is only a signal power measurement, the corresponding geometric loss is 1.14 dB using the weighted 32-channel geometric loss. Finally, the expected waveguide propagation loss on chip from 2 dB/cm loss characterized by foundry is 0.321 dB. De-embedding these losses from the measurement, the measured antenna insertion loss is 3.82 dB, compared to the simulated antenna insertion loss of 3.78 dB. The cause of this minute discrepancy between measurement and simulation can be due to the difference in waveguide propagation loss instead of exactly 2 dB/cm. The splitters in the antenna make up 1.12 dB (4 × 0.28 dB from

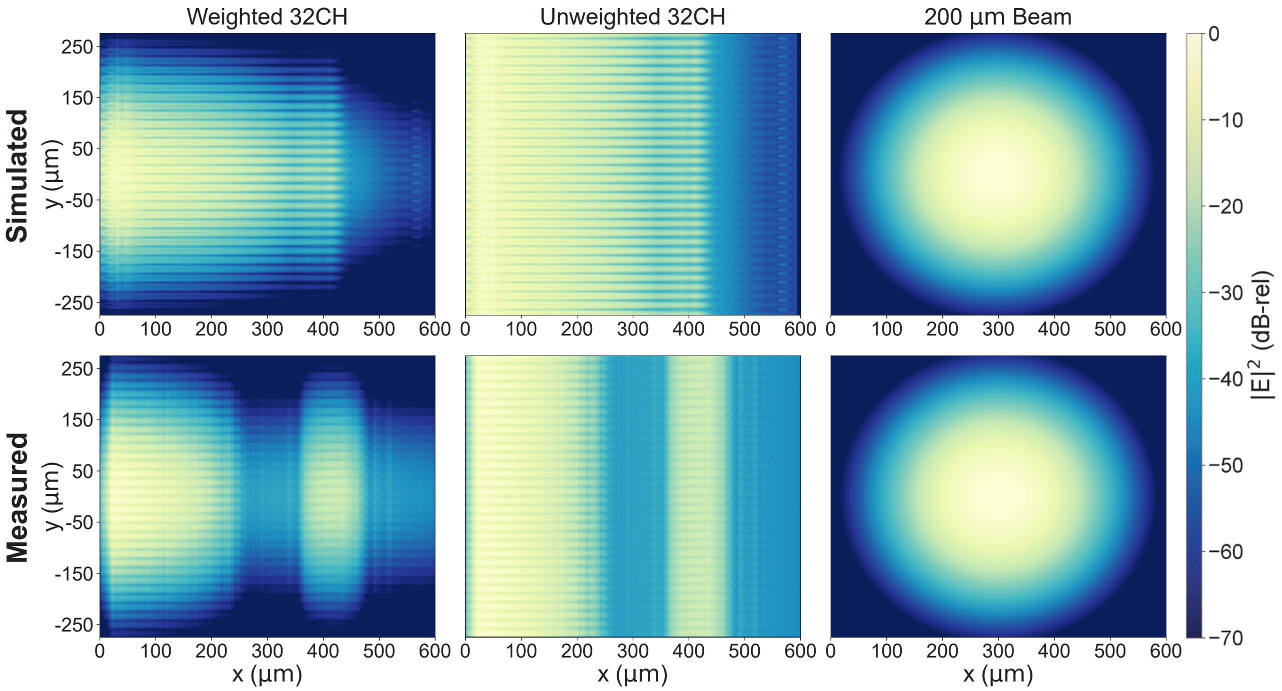

SUPPLEMENTARY FIG. S7. Near-field scattering profiles for weighted 32-channel aperture (left), unweighted 32 channel aperture (middle), and 200 μm beam diameter Gaussian beam (right) constructed from simulated (top) and measured (bottom) near-field radiation patterns.

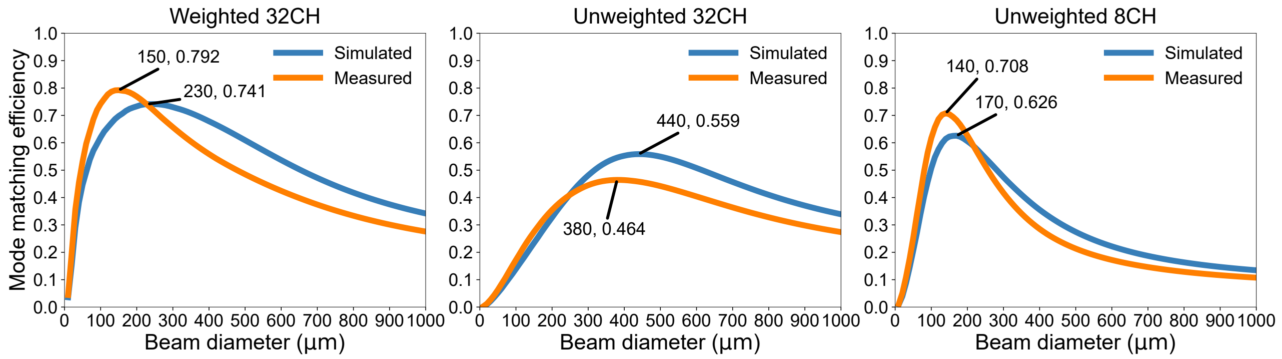

SUPPLEMENTARY FIG. S8. Geometric efficiencies of the weighted 32 channel (left), unweighted 32 channel (middle), and unweighted 8 channel (right) aperture profiles with Gaussian collimated beams of varying beam diameters, characterizing geometric loss.

[1]) of this loss, leaving 2.70 dB measured loss from the downward scattering and propagation loss in the active area. This loss can be attributed to 0.11 dB loss from the propagation loss in the active area using the 2 dB/cm specification and 2.59 dB loss from downward scattering. Therefore, the majority of the insertion loss is from the downward scattering that arises due to the vertically symmetric geometry of the MMA. Using multiple etch layers or adding back-reflectors to the antenna ([2–5]) can reduce this loss significantly in future MMA designs.

B. Quantum coherent receiver

The quantum(-limited) coherent receiver (QRX) comprises a tunable Mach-Zehnder interferometer (MZI) feeding into a balanced Ge photodiode pair followed by an electronic transimpedance amplifier (TIA). The QRX needs to coherently downconvert and amplify the quantum optical signal while minimizing decoherence with as high timing resolution as possible. This goal introduces several design strategies for QRX design.

1. QRX design

The performance of a QRX in acquiring quadrature information of a quantum optical state is characterized by its insertion loss, common-mode rejection ratio (CMRR), shot noise clearance (SNC), LO power knee (P_{knee}), 3-dB bandwidth (BW_{3dB}) and shot-noise limited bandwidth (BW_{shot}) [6–9]. The output noise power of a QRX can be referred to the input of the electronics (i_q^2) and is, to first order, proportional to

$$i_q^2 \propto L^2 P_{LO}\left(1 + \frac{\text{RIN}P_{LO}}{4\text{CMRR}}\right) + i_n^2 \tag{S3}$$

where L signifies the detection efficiency of the optical receiver, including the responsivity of the photodetectors for a given quantum efficiency and the optical efficiency for a given optical path loss. P_{LO} is the LO power proportional to the LO mean photon number. RIN is the relative intensity noise of the LO, which becomes RIN $= 1/P_{LO}$ for shot-noise-limited LO. CMRR is the common-mode rejection ratio defined as $P_{unb}/4P_{bal}$, where P_{unb} is the measured RF power when the QRX is unbalanced (when the optical power split between the photodiodes in the balanced detector is 100:0), and P_{bal} is the measured RF power when the QRX is balanced (when the optical power split between the photodiodes in the balanced detector is 50:50). Lastly, i_n is the input-referred current noise of the electronics. A more convenient expression can be derived by dividing by P_{LO}.

$$\frac{i_q^2}{P_{LO}} \propto L^2\left(1 + \frac{\text{RIN}P_{LO}}{4\text{CMRR}}\right) + \frac{i_n^2}{P_{LO}} \tag{S4}$$

The first term of this expression is the signal shot noise, the second term is the noise contribution from LO, and the third term is the noise contribution from the electronics. The CMRR sets the noise contribution from LO compared to signal. Assuming the QRX is operated in the signal shot-noise-limited regime with a high enough CMRR, the output noise power will increase linearly with LO power. At low CMRR, this linear relationship breaks down, introducing decoherence to the downconverted signal [7]. The shot noise clearance is defined as the ratio between the total noise power at the maximum LO power ($P_{LO,max}$) and the noise power at no LO power, assuming the QRX is operated in the signal shot-noise-limited regime with a high enough CMRR [7].

$$SNC \propto \frac{L^2 P_{LO,max}}{i_n^2} + 1 \tag{S5}$$

Maximizing the shot noise clearance is crucial to minimize decoherence introduced to the signal. This metric also sets the signal-to-noise ratio (SNR) in the classical operation of the QRX. LO power knee is defined as the LO power required to have 3 dB shot noise clearance (at which the QRX is shot noise limited).

$$P_{knee} \propto \frac{i_n^2}{L^2} \tag{S6}$$

The P_{knee} should be minimized to reduce the QRX power consumption and ensure enough LO power can be supplied to the system as more QRXs are integrated on-chip. The 3-dB bandwidth sets the classical operation bandwidth of a QRX. Due to the frequency-dependent response of the QRX, L can be defined as a function of frequency, $L(f)$. The frequency at which loss increases by 3 dB is defined as the 3-dB cut-off frequency, namely $L(f_{3dB}) = L(0)/2$. At the 3-dB cut-off frequency, the QRX output signal will be attenuated by 3 dB. However, in the infinite SNC limit, this 3-dB attenuation would not introduce decoherence to the quantum state and, for classical operation, does not change the SNR. Shot-noise-limited bandwidth is the bandwidth at which the SNC is reduced to 3 dB and at which the QRX stops being shot-noise-limited. Similar to 3-dB bandwidth, for a frequency dependent L, the shot-noise-limited cut-off frequency is defined as the frequency at which $P_{knee} = P_{LO,max}$, namely $L(f_{shot}) = i_n^2/P_{LO,max}$. Above this frequency, significant decoherence is introduced to the signal. Therefore, this is the bandwidth used for the quantum operation of the QRX.

The MZI and balancing of the photodiodes are crucial to ensure high CMRR. A simplified circuit diagram of the tunable interferometer and the CMRR auto-correction circuit is shown in Fig. S9a. In the case of MZI, the tuning range is set by the ideal 50:50 coupling of the directional coupler pair and the maximum relative phase shift that the push-pull thermo-optic phase shifter can introduce to the branches. While in this case, MZI was designed to have a full tuning range (from 0:100 to 100:0 power coupling), a more compact QRX can be realized in future designs by setting the tuning range to be as much as the variance in the imperfections in the 50:50 coupling caused by fabrication variations. The phase shifters in the MZI use resistive heaters made out of doped Si with 1 kΩ resistance in series with 1 V forward voltage Si diodes to enable push-pull configuration. The push-pull configuration allows both positive and negative voltages to be used by turning on/off heaters in the branches. The MZI is designed to provide sufficient tuning with ±5 V drivers. The measured IV characteristic of the push-pull phase shifter from -5 V to 5 V is shown in Fig. S9b. The forward voltages of the diodes in heaters 1 and 2 are 0.921 V and 0.911 V, respectively. The half-wave power of the MZI is measured by tuning the MZI from balanced (50:50) to unbalanced configuration (100:0). The test structure MZI reaches balanced configuration at -2.70 V and unbalanced configuration at 4.20 V. This corresponds to a half-wave power of 18 mW using the measured IV characteristic. Therefore, the optical power splitting for the ideal MZI without fabrication variations can be

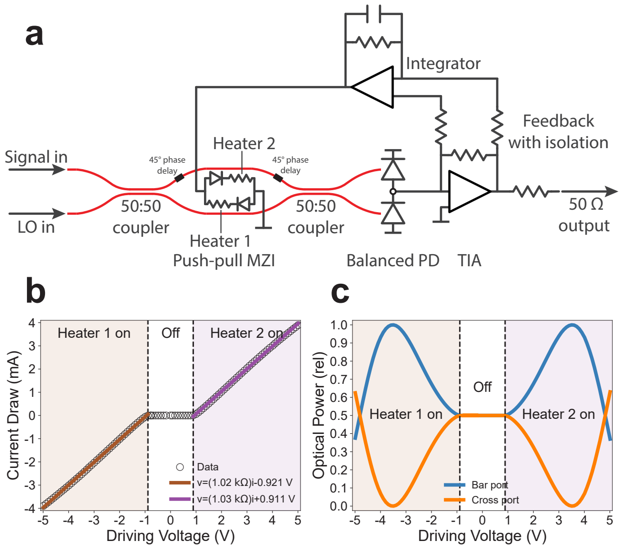

SUPPLEMENTARY FIG. S9. a) Simplified circuit diagram of the QRX with CMRR auto-correction. An error signal is extracted by probing the DC current through the TIA feedback resistor. The resulting voltage is sent to an integrator op-amp isolated from the TIA. The output voltage from the integrator is sent to the push-pull MZI to automatically correct the DC offset current from the balanced PD. The polarity of the feedback circuit ensures the MZI is pushed toward 50:50 coupling when LO power is higher than signal power. b) IV characteristic of the push-pull TOPS, comprising resistive heaters with series diodes. Either one of the heaters is turned on depending on the polarity of the driving voltage by turning on/off the series diodes. Linear regression fits are applied to the data points when the diodes are on to extract 1.02 kΩ and 1.03 kΩ resistance for heater 1 and heater 2, respectively. The forward voltages for the diodes in heater 1 and heater 2 are 0.921 V and 0.911 V, respectively. c) MZI response of the push-pull MZI constructed from the measured half-wave power of 18 mW and the measured IV characteristic.

simulated using the half-wave power and the IV characteristic, as shown in Fig. S9c. In the design, the photodiodes should also have the same quantum efficiencies and time constants to ensure high CMRR at every frequency. The reverse bias of the photodiodes can be tuned to match their time constants. However, increasing the reverse bias will increase the dark current that could decrease SNC and increase P_{knee}. Therefore, both MZI and photodiodes should be co-designed to ensure maximum CMRR while not compromising other specifications. Finally, the CMRR auto-correction circuitry described in the main text can be used to realize high-CMRR coherent receivers at scale.

In addition to CMRR, SNC and P_{knee} are set by the dark current of the photodiodes and the electronic noise floor of the electronics. The dark current of the photodiodes can be minimized by lowering the reverse bias, but this introduces a trade-off with bandwidth. Apart from dark current, TIA design is crucial to minimize the input-referred current noise of the electronics. The TIA should provide enough gain to lower the noise contribution of the subsequent electronics and external noise sources, such as electromagnetic interference (EMI), while contributing as minimal noise as possible itself. The noise contribution of the TIA is fundamentally limited by the thermal noise of the resistance determining the transimpedance gain, which scales as $i_n = \sqrt{4kT/R}$, where i_n is the input-referred current noise spectral density, k is the Boltzmann constant, T is the temperature, and R is the resistance. Therefore, increasing the resistance is crucial to maximize SNC and minimize P_{knee}. Since the gain-bandwidth product (f_t) of the transistors is set by the process technology, this resistance, and consequently electronic noise, has a fundamental trade-off with the bandwidth.

Since sensing and communication applications have different specification requirements from a QRX, two configurations are designed to meet the demands of both. The high bandwidth configuration utilizes a low-gain but high-bandwidth TIA to optimize for communications and the high shot noise clearance configuration utilizes a high-gain but low-bandwidth TIA to optimize for sensing. We note that both configurations use the same photonic design but different electronic TIA designs. The 32-channel phased array system uses the TIA used in the high shot noise clearance configuration. Therefore, the high shot noise clearance QRX represents a single channel of the 32-channel QRX in the system.

2. *Insertion loss characterization*

Optical insertion loss of QRX PIC was characterized by sending power to the LO port and measuring the total photocurrent from the PDs. For 136 μW input power, the measured photocurrent was 44.0 μA, corresponding to 4.88 dB loss. The standard grating coupler that couples LO power into QRX has 3.30 dB loss. De-embedding this loss, QRX has an optical insertion loss of 1.58 dB. PDs have a quantum efficiency of 70.4%, corresponding to 1.52 dB loss. Waveguide propagation loss for the LO through 300 μm path in the single QRX test structure is 0.0599 dB from 2 dB/cm measured path loss with negligible excess loss from the phase shifter and couplers in the MZI. This leads to an expected optical insertion loss of 1.58 dB that matches well with the measurement.

3. *Common-mode rejection ratio characterization*

Since the CMRR is set by the QRX PIC, both high BW and high SNC configurations have the same CMRR. CMRR is measured by setting the push-pull MZI in the PIC to unbalanced (100:0) and balanced (50:50) settings while injecting intensity-modulated LO to the QRX. A 1550 nm source (APEX AP3350A) is intensity modulated at 1.1 MHz, and the modulated light is sent to the LO port of the QRX. The QRX

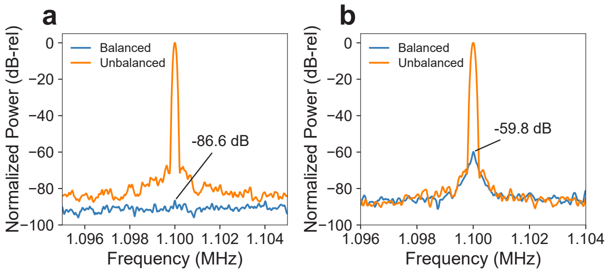

SUPPLEMENTARY FIG. S10. a) Measured averaged spectra when the MZI is set to balanced and unbalanced configurations with the same LO power showing a peak amplitude difference of 86.6 dB limited by the dynamic range of the ESA. b) Measured averaged spectra when the MZI is set to balanced and unbalanced configurations with 18.2 dB higher LO power for the balanced configuration measurement. The peak amplitude difference is 59.8 dB, corresponding to a CMRR of 59.8 dB+2*18.2 dB-6 dB=90.2 dB.

output is connected to an RF signal analyzer to measure the RF power at 1.1 MHz. Two measurements are taken by tuning the MZI to unbalanced and balanced settings. Same as the definition in Section I B, CMRR in dB is then calculated as

$$\mathrm{CMRR} = 10\log_{10}\left(\frac{P_{unb}}{4P_{bal}}\right) \tag{S7}$$

where P_{unb} and P_{bal} are the unbalanced and balanced RF power measurements, respectively. The CMRR measurements were taken with the signal analyzer using a frequency span of 10 kHz, resolution and video bandwidths of 100 Hz, and a center frequency of 1.1 MHz. To validate the stability of CMRR, ten snapshots were taken over the course of 10 seconds for both balanced and unbalanced settings. The CMRR was then calculated from the average of the traces in these snapshots. In the initial measurement, due to the limited dynamic range of the ESA, the balanced measurement was able to suppress the 1.1 MHz peak below the noise floor as seen in Fig. S10a. The average CMRR from 10 traces measured over 10 seconds from this initial measurement is 80.6 dB. To increase the dynamic range and resolve the suppressed peak, the LO optical power in balanced measurement was increased by 18.2 dB. The LO optical power difference between balanced and unbalanced measurements was monitored and recorded throughout the measurements with an average power difference of 18.2 dB. Since the change in RF power has a quadratic relation with the change in optical power, we add double the optical power difference in dB to the measured peak amplitude difference. The resulting traces after changing the LO power in the balanced measurement are shown in Fig. S10b. The average CMRR from 10 traces measured over 10 seconds from this final measurement is 90.2 dB, with a maximum CMRR of 92.3 dB. This CMRR was measured for only a single frequency of 1.1 MHz but can be repeated for other frequencies to measure the CMRR response as well as will be shown in Sec. II A.

4. High shot noise clearance configuration

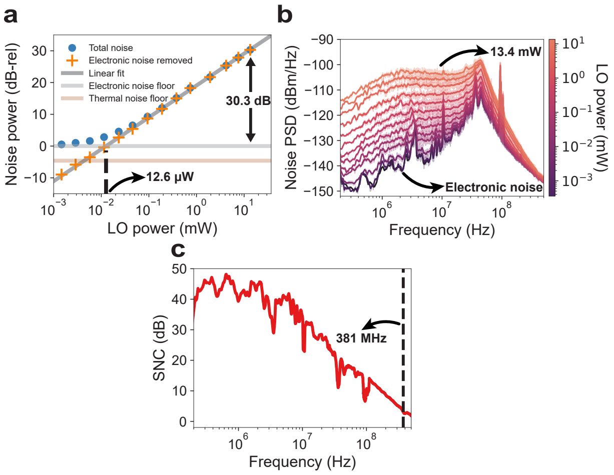

SUPPLEMENTARY FIG. S11. a) SNC curve of the high-SNC QRX showing 30.3 dB SNC and 12.6 μW P_{knee} along with the measured electronic noise floor and the theoretical thermal noise floor. A linear regression fit is applied to the measured data to show the QRX is signal shot noise limited with its noise power increasing linearly with LO power, showing a near-unity gradient of 1.004±0.006. b) Measured noise spectra for varying LO powers showing raw data (semi-transparent lines) and processed data with a moving average filter (solid lines). c) Measured SNC spectrum of the high-SNC QRX showing a shot-noise-limited bandwidth of 381 MHz.

In the high SNC configuration, the QRX PIC is packaged with a discrete TIA (LTC6269-10) on a custom PCB. As described in Methods, the discrete TIA is a FET-input op-amp with resistive feedback. The op-amp IC has a 4 GHz gain-bandwidth product and is used with a 50 kΩ feedback resistor. The capacitance of the feedback trace is used to ensure sufficient phase margin while keeping the closed-loop gain greater than 10 since the op-amp is decompensated. A 50 Ω resistor is placed in series with the output of the TIA for impedance matching and to dampen any oscillations from capacitive loading at the output. The TIA output is routed with 50 Ω coplanar waveguide transmission lines to an SMA port. The SMA port is then connected to an RF signal analyzer. The balanced PDs of the high SNC QRX are biased to 0 V to minimize the dark current and the noise coupling through the bias circuit, resulting in a PD bandwidth of >10 GHz.

Similar to high BW QRX, SNC and P_{knee} characterization is done by sending LO to the LO port of the QRX and sweeping the power. The output is sent to an ESA with a resolution bandwidth of 10 kHz and a video bandwidth of 1 kHz. The noise spectra of the high SNC QRX with different LO powers are

seen in Fig. S11b. A moving average filter is applied to the raw data (transparent lines) to construct the processed data (solid lines). These noise spectra are taken at total photocurrents ranging from 0 mA to 9.33 mA, corresponding to 13.4 mW maximum LO power above which the PDs saturate due to the carrier screening effect. The variation in this saturation current between the two configurations can be attributed to the fabrication variations between the PDs in the two PICs. Since this measurement was not taken in a Faraday cage, there are tones in the spectrum due to EMI from RF transmitters nearby. The tones at 100 MHz are from the broadcast stations on Mount Wilson near Pasadena.

The output noise powers integrated over the QRX bandwidths normalized to the electronic noise power as a function of LO power are shown in Fig. S11a. LO power is determined by measuring the total photocurrent from the PDs and de-embedding the characterized QRX insertion loss (1.58 dB). The electronic noise floor along with the thermal noise floor resulting from 50 kΩ are also shown with a difference in noise powers of 4.60 dB. This higher difference can be attributed to external noise such as EMI coupling into QRX. The shot noise powers are also plotted by subtracting the electronic noise powers from the total noise powers, and a linear regression fit is applied to the points above the 3 dB SNC. The gradient from the fit is near unity at 1.004±0.006, confirming the QRX is operating at the signal shot noise limited regime. The QRX has an SNC of 30.3 dB over its bandwidth and is shot-noise limited at 8.72 μA photocurrent, corresponding to a P_{knee} of 12.6 μW. BW_{shot} is characterized by dividing the noise spectrum at maximum LO power by the electronic noise spectrum, resulting in the SNC spectrum shown in Fig. S12c. The SNC drops at 89.8 MHz due to the EMI from Mount Wilson FM transmitters. The SNC rises again and drops to 3 dB at a BW_{shot} of 381 MHz.

5. *High bandwidth configuration*

In the high bandwidth configuration, an integrated TIA (ONET4291T) is packaged with a single QRX PIC [10]. The integrated TIA is a three-stage amplifier with two gain stages and a differential output buffer stage. It has a transimpedance gain of 3.2 kΩ and a 3-dB bandwidth of 2.8 GHz[11]. Any parasitics introduced in the path between the photodiodes and the TIA can distort the frequency response of the QRX and reduce bandwidth. Therefore, the bare dies are placed as close as possible with a wirebond less than 200 μm length between the PD output and TIA input. The balanced PDs of the high BW QRX are biased to -1 V to maximize the bandwidth without significantly sacrificing SNC, resulting in a PD bandwidth of >15 GHz. The co-packaged photonic-electronic integrated circuit is mounted on a custom PCB with wirebonds to the chip pads. The differential outputs from the TIA are wirebonded to two 50 Ω matched coplanar waveguide transmission lines on the PCB that are routed to two SMA ports. The packaged die photo along with a simplified schematic of the QRX is shown in Fig. S12a.

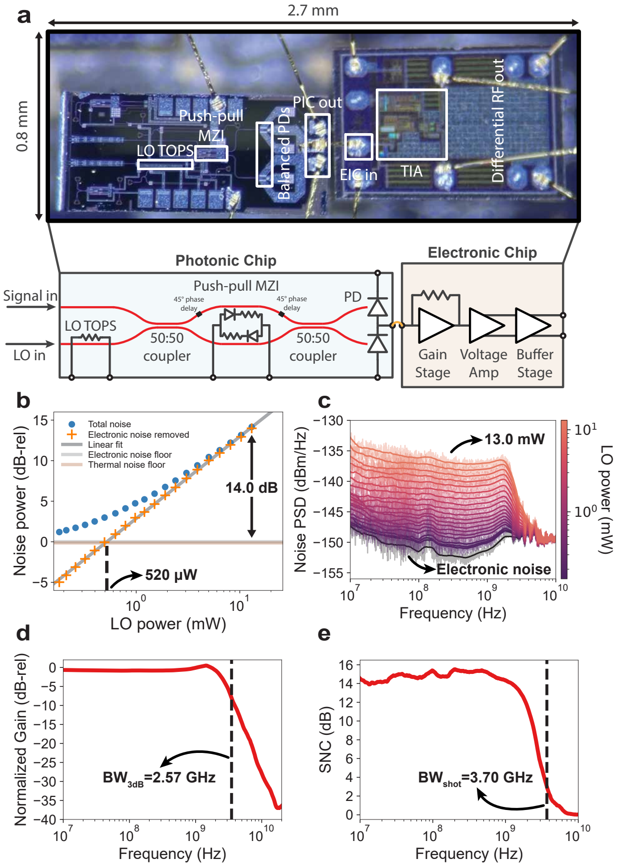

SUPPLEMENTARY FIG. S12. a) The packaged die photo and the simplified schematic of the high-BW QRX. b) SNC curve of the high-BW QRX showing 14.0 dB SNC and 520 μW P_{knee} along with the measured electronic noise floor and the theoretical thermal noise floor. A linear regression fit is applied to the measured data to show the QRX is signal shot noise limited with its noise power increasing linearly with LO power, showing a near-unity gradient of 1.009±0.016. c) Measured noise spectra for varying LO powers showing raw data (semi-transparent lines) and processed data with a moving average filter (solid lines). d) Measured optoelectronic gain of the high-BW QRX showing a 3-dB bandwidth of 2.57 GHz. e) Measured SNC spectrum of the high-BW QRX showing a shot-noise-limited bandwidth of 3.70 GHz.

The high bandwidth QRX is characterized to determine its BW_{3dB}, BW_{shot}, SNC, and P_{knee}. BW_{3dB} is characterized by a 20 GHz vector network analyzer (Keysight N5230A). A 1550 nm source (Pure Photonics PPCL700) is modulated by a 40 GHz LiNb modulator (Thorlabs LNA6213) driven by the VNA. S parameters of the modulator are collected with a 40 GHz InGaAs photodiode (Optilab PD-40-M-AC) and de-embedded from the final measurement. The differential outputs from the QRX are connected to the VNA, and the S parameters of the output paths

are also de-embedded. After calibration, the modulated light is sent to the LO port of the QRX, and the frequency response of the QRX is acquired with a 3-port optoelectronic S-parameter measurement. As seen in Fig. S12d, packaged QRX has an optoelectronic BW_{3dB} of 2.57 GHz.

SNC and P_{knee} characterization is done by sending LO to the LO port of the QRX and sweeping the power. One of the differential outputs is connected to an ESA to measure the noise floor, and the photocurrents from the PDs are monitored with high-precision current meters as the LO power is swept. LO power is determined by measuring the total photocurrent and de-embedding the characterized QRX insertion loss (1.58 dB). The ESA is set to a resolution bandwidth of 300 kHz and a video bandwidth of 3 kHz. The measurements are also done in a Faraday cage to minimize RF interference from external sources. The noise spectra of the high BW QRX with different LO powers are seen in Fig. S12c. A moving average filter is applied to the raw data (transparent lines) to construct the processed data (solid lines). These noise spectra are taken at total photocurrents ranging from 0 mA to 9.04 mA, corresponding to 13.0 mW maximum LO power above which the PDs saturate and distort the spectrum due to nonlinearity from the high amount of photo-excited carriers in the junction [12]. The SNC and P_{knee} can be extracted from this measurement by integrating the noise spectrum over the operating bandwidth of the QRX. Output noise powers integrated over the QRX bandwidths normalized to the electronic noise power as a function of LO power is shown in Fig. S12b. Electronic noise floor along with the thermal noise floor resulting from 3200 Ω are also shown with a difference in noise powers of 0.1 dB, showing that the high BW QRX is operating at the fundamental thermal noise limit. Shot noise powers are also plotted by subtracting the electronic noise powers from the total noise powers, and a linear regression fit is applied to the points above the 3 dB SNC. The gradient from the fit is near unity at 1.009±0.016, confirming the QRX is operating at the signal shot noise limited regime as needed. QRX has an SNC of 14.0 dB over its bandwidth and is shot-noise limited at 0.362 mA photocurrent, corresponding to a P_{knee} of 520 μW. BW_{shot} is characterized by dividing the noise spectrum at maximum LO power by the electronic noise spectrum, resulting in the SNC spectrum shown in Fig. S12e. The SNC drops to 3 dB at 3.70 GHz, setting the bandwidth over which the high bandwidth QRX is shot noise limited. As seen here, the higher bandwidth leads to a lower SNC compared to the lower bandwidth but higher SNC of the high SNC QRX.

C. Thermo-optic phase shifter

Thermo-optic phase shifters used to modulate LO phase use TiN resistive heaters 2 μm above the Si waveguides. The waveguide in the TOPS is 300 μm long and the resistance of the heater is 630 Ω, designed to have a full 2π phase shift with ±5 V drivers. The IV characteristic of the TOPS is shown in Fig. S13a. A linear regression fit is applied to the data, showing a resistance of 566 Ω. The optical response of the TOPS was measured with an MZI test structure. The MZI test structure comprises a standard grating coupler for optical input, a Y-junction splitter, two TOPS, a Y-junction combiner, and another standard grating coupler for optical output. To account for the first-order thermal crosstalk, TOPS spacing in the MZI is the same as the LO TOPS spacing in the PIC (67 μm). By driving one of the TOPS in the MZI, the relative phase shift between two channels was characterized. To have a full 2π optical power swing at the output grating coupler, 0 V to 4.37 V ramp modulation was applied. Using the measured IV characteristic, this corresponds to a half-wave power (P_π) of 17.0 mW. Using this P_π, the MZI response and the relative phase shift between two LO channels as a function of driving voltage can be simulated as shown in Fig. S13b. Finally, the 3-dB bandwidth of the TOPS was characterized by sweeping the modulation frequency and was measured to be 11.4 kHz.

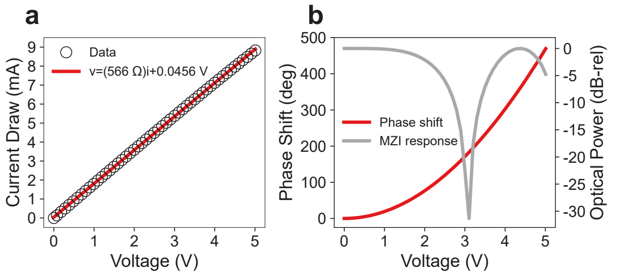

SUPPLEMENTARY FIG. S13. a) IV characteristic of the TOPS with a linear regression fit showing 566 Ω resistance. b) MZI response and the corresponding phase shift of the TOPS constructed from a half-wave power of 17.0 mW.

II. PIC SYSTEM CHARACTERIZATION

After component characterization, the PIC was packaged with electronics (high SNC configuration) on an RF motherboard as described in the system electronics section of the Methods. The pictures of the packaged system are shown in Fig. S1.

Scaling quantum systems is a non-trivial and challenging task that serves as the bottleneck for many quantum technologies. The phased array system was characterized to show it can operate at scale with a high number of parallelized channels with minimal crosstalk, parasitics and noise as the system is scaled. A key benchmark for this is to confirm all of the QRX channels are working in the signal shot-noise-limited regime with high CMRR and SNC. Therefore, before the experiments, all of the 32 channels were charac-

terized to find their CMRR and SNC.

A. 32-channel common-mode rejection ratio (CMRR)

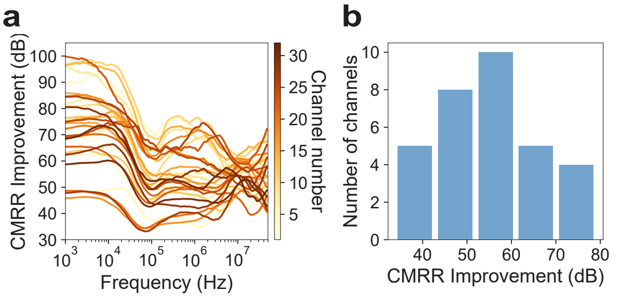

SUPPLEMENTARY FIG. S14. a) Measured CMRR improvement spectra of all 32 channels setting the lower bound on their CMRRs. b) Histogram of the maximum CMRR improvements showing a median of 76.8 dB, a minimum of 52.4 dB, and a maximum of 104.2 dB.

CMRR auto-correction described in Sec. I B allows the system to work at scale with quantum-limited sensitivity and protects the system from fabrication variations as the QRX channels are arrayed. The increase in CMRR in all channels between when the CMRR auto-correction is turned off and on was characterized by sending an intensity modulated LO into the PIC. 32 channel outputs were connected to 32 digitizers with 100 MHz bandwidth and 100 MSa/s sampling rate. 10 ms traces were recorded with CMRR auto-correction turned off and on. The amplitude modulation frequency was swept from 1 kHz to 50 MHz to characterize the CMRR increase of all 32 channels with CMRR auto-correction at every operating frequency. These traces were then converted into frequency domain, and the peak amplitudes at the modulation frequencies were recorded. The change in amplitude between when the CMRR auto-correction is turned off and when it is turned on characterizes the improvement in CMRR for each channel with CMRR auto-correction. While this doesn't directly characterize CMRR, it gives a lower bound on the CMRR of each QRX. In other words, each QRX channel must have an equal or higher CMRR than its respective CMRR improvement. The resulting improvements in CMRR over frequency are shown in Fig. S14a. To characterize the variation in CMRR across channels, the maximum CMRR improvement for each trace is recorded and plotted in a histogram in Fig. S14b. The median CMRR improvement is 76.8 dB. with a minimum of 52.4 dB and a maximum of 104.2 dB.

B. 32-channel shot noise clearance (SNC)

High SNC QRX design enables arrayed coherent receivers with quantum-limited sensitivity at scale. Due to its low P_{knee}, a large number of channels can be parallelized operating at the signal shot noise limited regime without hitting a bottleneck in available LO power. In addition to reducing power consumption, the 12.6 μW P_{knee} allows thousands of QRX channels to be parallelized with a single LO optical input before hitting the two-photon absorption power ceiling in Si waveguides [13]. To confirm high SNC operation of QRX at scale, SNCs of all 32 channels were recorded with the ESA. Similar to the SNC characterization in Sec. I B, noise spectra of the channels were acquired with the PIC being injected with 49.3 mW LO power corresponding to, on average, 1.54 mW LO power per QRX. The same noise spectra were also acquired without any LO power to measure the electronic noise floor. The ratio between the two noise spectra gives the SNC frequency response. Measured SNC frequency responses of all 32 QRXs are shown in Fig. S15a. As seen here, all channels operate well into the signal shot noise limited regime (SNC>10 dB). To characterize the variation in SNC across QRXs, the maximum SNC over the spectrum without integrating over bandwidth is recorded for each QRX and plotted in a histogram as shown in Fig. S15b. The maximum SNC variation is low, with a minimum of 25.3 dB, a maximum of 27.7 dB, and a median of 26.6 dB. This variation could be caused by the varying LO powers, varying electronic noise floors and varying frequency responses across the channels. We note that the lower median SNC here is caused by the fact that LO power is less than the LO power sent in the single channel QRX characterization due to the PDs in one of the QRXs here saturating at a lower LO power than the single channel QRX.

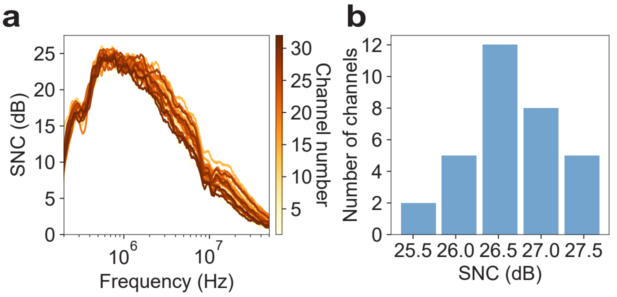

SUPPLEMENTARY FIG. S15. a) Measured SNC spectra of all 32 channels showing shot-noise-limited operation across all QRXs. b) Histogram of the SNCs showing a minimum of 25.3 dB, a maximum of 27.7 dB, and a median of 26.6 dB.

III. ON-CHIP SQUEEZING ANALYSIS

Here we demonstrate how we extract the squeezing and antisqueezing levels for the experiments in Fig. 5 of the main text. The analysis for the 32-channel source characterization data of Fig. 5c is shown in Fig. S16. For each pump power (P) of the 32-channel

sweep, we measure noise power traces for the squeezed vacuum and vacuum states using an RF spectrum analyzer with a 2 MHz RBW, 5 Hz VBW, and 1.25 kHz sampling rate. Each vacuum trace is taken immediately before or after a squeezed vacuum trace. Due to random phase drifts, we acquire multiple pairs of vacuum and squeezed vacuum traces until an approximately uniform sampling of phases is achieved. For a pair of vacuum and squeezed vacuum traces, each trace is acquired over the same amount of time, typically over 5-10s. To correct long-term drifts in the LO power, each pair is normalized by the mean of the vacuum trace. The normalized traces are concatenated to obtain squeezed vacuum and vacuum traces corresponding to an approximately uniform phase distribution. To perform the estimation procedure on the same number of points for all pump powers, 10^4 points are randomly sampled from each concatenated trace. The histograms are constructed for the sampled noise powers, and the PDFs are approximated by kernel density estimation (Fig. S16). The squeezing and antisqueezed level estimates are obtained from the peaks in the derivative of the KDEs. The KDEs are compared with a theoretical model for the PDFs with a uniform phase distribution. The theoretical models are calculated with the experimental shot noise distribution standard deviation of $\sigma = 0.012$ dB, $\eta = 0.0157$, and $r = \mu\sqrt{P}$, where $\mu = 0.038$ $[\mathrm{mW}]^{-1/2}$ (see Sec. IV). The experimental estimates (blue), theoretical estimates (purple), and the noise floor (black) are indicated with dashed lines in Fig. S16. The experimental and theoretical estimates are in near agreement (well within σ) despite the phase noise in the measurements. We note that some discrepancies in the theoretical and experimental estimates are expected because the model fit was performed on the experimental estimates with only one free parameter across the entire data set rather than an individual fit to each histogram.

IV. SQUEEZED LIGHT SOURCE CHARACTERIZATION

The squeezed light sources are characterized with a modified version of the transmitter setup as shown in Fig. S17a. Continuous wave light from a fiber-coupled 1550 nm laser (OEWaves) is split into a signal path and a local oscillator (LO) path. The light in the signal path is sent to an erbium-doped fiber amplifier (PriTel EDFA) with a tunable gain. The amplified light is upconverted to 775 nm by second harmonic generation (SHG) with a periodically poled lithium niobate (PPLN) waveguide. The upconverted light is sent to another PPLN waveguide for Type 0 spontaneous parametric downconversion (SPDC) to generate squeezed light centered at 1550 nm. The squeezed light is sent to an isolator (Thorlabs), which rejects back-reflected light and serves as a filter for any residual 775 nm pump light. After the isolator, an optical switch is used to switch between the vacuum and squeezed vacuum state measurements. The squeezed light is sent to a polarization-maintaining 50:50 beamsplitter (BS) for interference with the LO.

In the LO path, the 1550 nm laser light is sent to an electro-optic phase modulator (EOSpace) to apply a phase ramp to the LO. The phase ramp is generated by sending a modulated signal from a function generator to the RF input of the modulator. After the modulator, a polarizing beamsplitter (PBS) removes light polarized along the fast axis to ensure interference in a single polarization mode at the beamsplitter. The outputs of the beamsplitter are sent to a fiber-coupled balanced homodyne detector (BHD). The BHD (Thorlabs PDB425C) has a 75 MHz bandwidth, 35 dB CMRR, and 1 A/W responsivity. The RF output of the BHD is sent to an RF spectrum analyzer (Keysight N9030b) operated in zero-span mode to measure the noise power levels in real time.

A total of four PPLN waveguides were used for SPDC in the experiments of the main text. The four experimental configurations are summarized in Table S1. For each configuration, a pump power sweep was performed to characterize the SPDC waveguide and the effective efficiency of the setup [14]. For SPDC, the waveguide-coupled 775 nm pump power (P) can be related to the squeezing parameter (r) by $r \approx \mu\sqrt{P}$ [15], where μ is the SPDC efficiency [16]. The amount of squeezing can be characterized experimentally by comparing the variance of the quadratures measured with a squeezed state input to that measured with a vacuum state input in homodyne detection,

$$\frac{\langle \Delta\hat{Q}(\phi)^2\rangle_{\mathrm{sq}}}{\langle \Delta\hat{Q}(\phi)^2\rangle_{\mathrm{vac}}} = \eta(e^{-2r}\cos^2\phi + e^{2r}\sin^2\phi) + 1 - \eta. \tag{S8}$$

From Eq. S8, the squeezing (ΔQ_-^2) and antisqueezing (ΔQ_+^2) levels relative to the shot noise level in terms of the pump power are,

$$\Delta Q_\pm^2 = \eta \exp\Big(\pm 2\mu\sqrt{P}\Big) + 1 - \eta, \tag{S9}$$

where η is the effective efficiency, including the effects of source loss, fiber-optic loss, detector loss, and shot noise clearance. For each pump power, the noise levels for the squeezing vacuum and vacuum states were measured over multiple LO phases. The pump power was varied by tuning the gain of the EDFA. To characterize the SPDC waveguide in configuration 1, an EDFA with up to 27 dBm output power was used, and to characterize the SPDC waveguides in configurations 2-4, an EDFA with up to 37 dBm output power was used. A phase ramp was applied to the LO to ensure that noise power levels were accumulated over multiple periods. The squeezing and antisqueezing levels for all configurations were estimated from the noise power distribution using the procedure described in Section III.

The characterizations for the four sources are shown in Fig. S17b-e. The squeezing and antisqueezing es-

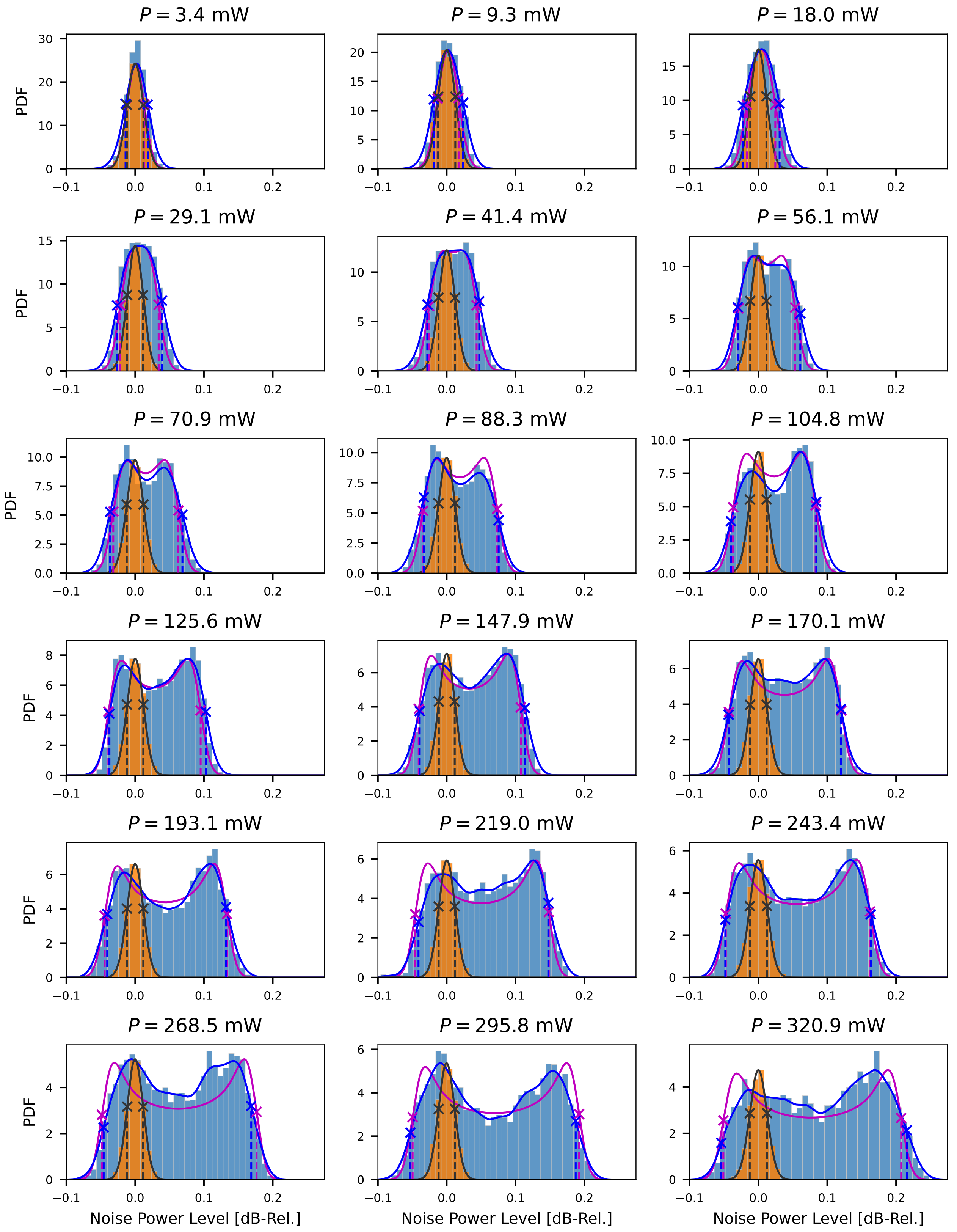

SUPPLEMENTARY FIG. S16. **32-channel source characterization**. Histograms of the sampled noise powers for the squeezed vacuum (blue) and vacuum (orange) states. The KDE for the squeezed vacuum histogram is plotted in blue, the theoretical model of the PDF for the squeezed vacuum histogram is plotted in purple, and a Gaussian fit to the vacuum histogram is plotted in black. The locations of the maximum slopes for the squeezed state KDE, vacuum state Gaussian, and theoretical model are indicated with crosses and dashed lines.

timates are plotted as a function of the waveguide-coupled power, which is calculated from,

$$P = \eta_{in} P_{\mathrm{SHG}}, \tag{S10}$$

where η_{in} is the 775 nm input coupling efficiency and P_{SHG} is the power measured at the output of the SHG. The red curves are the theoretical model for the squeezing and antisqueezing levels from Eq. S9. The best-fit parameters η and μ are obtained from a simultaneous least squares fit to the squeezing and antisqueezing levels. The errorbars are calculated from the Jacobian, and the residuals are evaluated at the optimal parameters. The key specifications for the four SPDC waveguides are summarized in Table S2. The fitted effective efficiencies η of Fig. S17b-e include the waveguide output coupling efficiencies η_{out} reported in Table S2 as well as all other system losses, which are delineated in Section VIII A.

Config.	SHG waveguide	SPDC waveguide	Experiments
1	Covesion H-spec.	HCP SC18068	Fig. 4c
2	HCP SC23399	Covesion H-spec.	Fig. 5b,c
3	HCP SC23399	HCP SC19075	Fig. 5f
4	HCP SC23399	Covesion M-spec.	Fig. 5e

SUPPLEMENTARY TABLE S1. Waveguide configurations used in the main text experiments. Configuration 1 was used for squeezed light imaging (Fig. 4c). Configuration 2 was used for beamforming channel sweep (Fig. 5b) and 32-channel pump power sweep (Fig. 5c). Configuration 3 was used for the field of view data (Fig. 5f). Configuration 4 was used for the beamwidth data (Fig. 5e).

SPDC waveguide	η_{in}	η_{out}	L (cm)	μ [mW]$^{-1/2}$
HCP SC18068	0.4	0.4	3	0.119
Covesion H-spec.	0.7	0.8	4	0.038
HCP SC19075	0.4	0.4	3	0.070
Covesion M-spec.	0.7	0.8	4	0.031

SUPPLEMENTARY TABLE S2. PPLN waveguides used as sources of squeezed light. η_{in} is the 775 nm input coupling efficiency, η_{out} is the 1550 nm output coupling efficiency, and μ is the SPDC efficiency obtained from a least-squares fit to the pump power sweep data in Fig. S17.

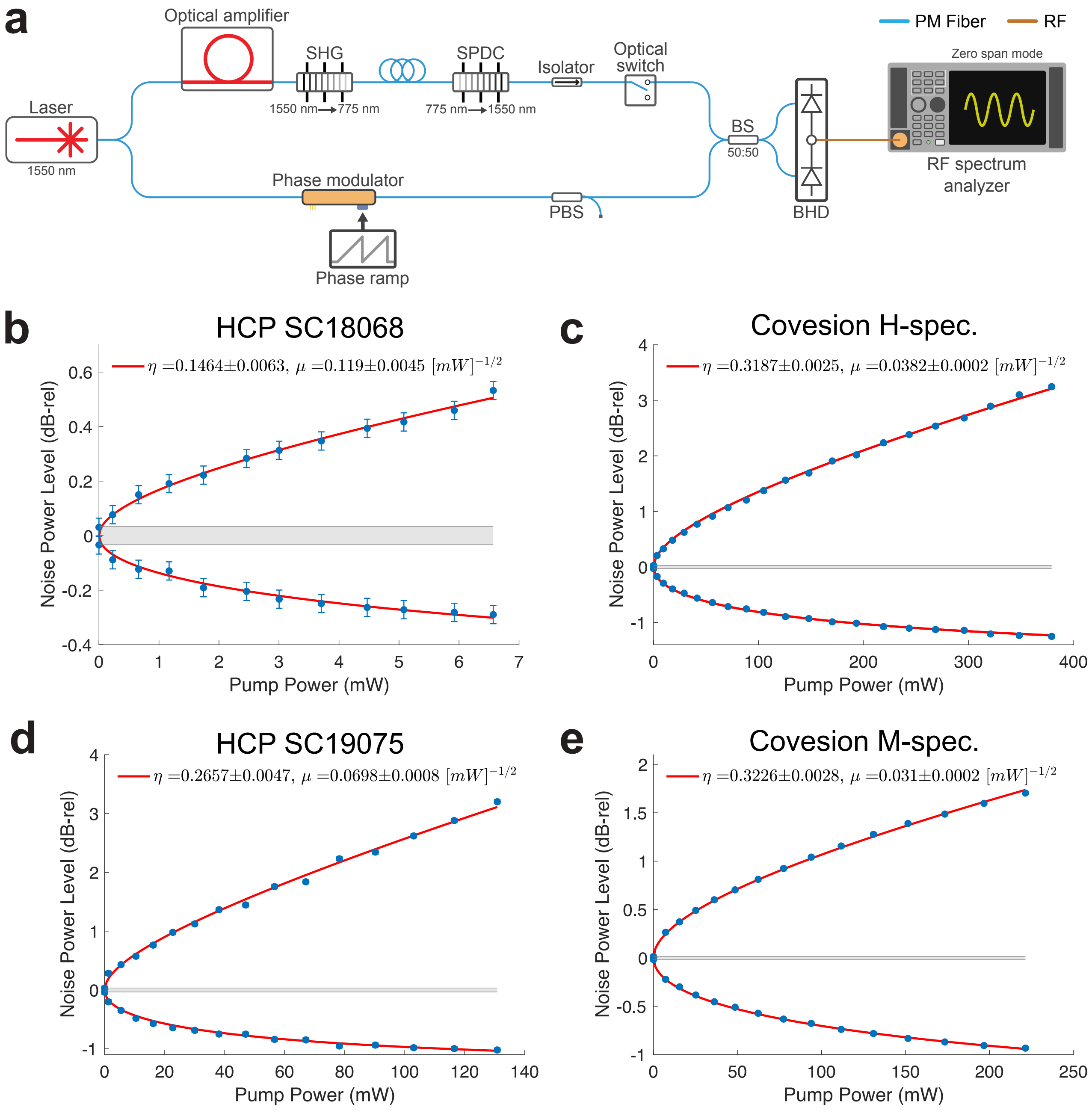

SUPPLEMENTARY FIG. S17. a) Experimental setup for source characterization. b) Configuration 1. Each noise power trace was measured over 10s with a sampling rate of 10 kHz, center frequency of 6 MHz, 1 MHz RBW, and 20 Hz VBW. c) Configuration 2. Each noise power trace was measured over 10s at 1 Hz phase modulation with a sampling rate of 5 kHz, 40 MHz CF, 8 MHz RBW, and 100 Hz VBW. d) Configuration 3. Each noise power trace was measured over 5s at 1 Hz phase modulation with a sampling rate of 20 kHz, 4 MHz CF, 20 MHz RBW, and 10 Hz VBW. e) Configuration 4. Each noise power trace was measured over 10s at 1 Hz phase modulation with a sampling rate of 10 kHz, 20 MHz CF, 8 MHz RBW, and 30 Hz VBW.

V. HIGH BANDWIDTH MEASUREMENT OF SQUEEZED LIGHT

To overcome the bandwidth limitation in measuring squeezing with the low-noise TIA used in the experiments and show the bandwidth advantage of photonic-electronic ICs for quantum technologies, a high BW QRX was used to measure squeezed vacuum up to its $\mathrm{BW_{shot}}$. The squeezed vacuum was generated in Configuration 4. Squeezed vacuum is injected into the high-BW QRX as signal and coherent light, phase modulated with a LiNbO phase modulator (EOSpace) at 1 Hz, with a continuous-wave power of 9.50 mW is injected into the high-BW QRX as LO. This LO power corresponds to an SNC of 12.7 dB seen in Fig. S12b. QRX output is connected to an RF signal analyzer (Keysight N9030B). The signal analyzer is used in zero-span mode with 8 MHz resolution bandwidth, 100 kHz video bandwidth, and varying center frequencies up to $\mathrm{BW_{shot}}$.

10-second snippets of the data measured at different frequencies ranging from 36 MHz to 5 GHz is shown in Fig. S18. The determined noise levels for squeezed and antisqueezed quadrature normalized to the shot noise level (SNL) with their respective error bars at each frequency are shown in Fig. 3d. Across the entire dataset, a maximum squeezing level of 0.15 ± 0.01 dB below the SNL and a maximum antisqueezing level of 0.52 ± 0.01 dB above the SNL was measured at a frequency of 366 MHz. From this estimate, we obtain $r = 0.661^{-0.041}_{+0.043}$ and $\eta = 0.046^{+0.005}_{-0.004}$. The setup used for this measurement was different than the experiments, and its loss wasn't rigorously characterized. Therefore, in addition to the QRX loss, additional losses affecting the detection efficiency can be alignment loss from fiber-to-chip coupling and additional fiber-optic loss in the tabletop setup.

VI. PHASE CALIBRATION ALGORITHM

Phase calibration for the chip utilizes a modified gradient search algorithm by sweeping phase settings of on-chip TOPS with an orthogonal mask set. Employing orthogonal mask sets to sweep the phase settings maximizes the dynamic range of the calibration to enhance phase accuracy [17]. The orthogonal mask set is set to be the unit vectors of the vector space of the phase shifter array. This results in sweeping the phase of each on-chip TOPS independently. Phase calibration is performed by sending 1550 nm light to the signal and applying a 5 MHz phase ramp to the LO. The peak power of the downconverted 5 MHz signal is measured with the ESA using a resolution bandwidth of 100 Hz and a video bandwidth of 1 Hz and is used as feedback information in the algorithm.

During phase calibration, TOPS voltage is swept, starting with large voltage steps and continuing with progressively smaller voltage steps with each optimization iteration. Due to the Gaussian amplitude profile, edge channels contribute less to the SNR of the combined output. Therefore, channel settings are swept starting from the edge channels and continuing to the middle channels. As each channel is tuned and the total SNR improves, the proportional increase in SNR from channel to channel gets smaller, leading to higher errors in the optimal phase setting of the latter channels that are swept. Therefore, for each optimization iteration, the order of channels to be swept is reversed. A pseudo-code for the phase calibration algorithm is shown in Algorithm VI.

Algorithm1 Phase calibration algorithm

$n_{ch} \leftarrow 32$ ▷ Number of phase shifter channels
$\Phi_{opt} \leftarrow \Phi_{init}$ ▷ Initialize phases
$P_{opt} \leftarrow 0$ ▷ Initialize measured power
LOAD(Φ_{opt}) ▷ Load initial phase settings
for $i \leftarrow 1$ to size(Φ_{steps}) **do**
  $\varphi_{step} \leftarrow \Phi_{steps}[i]$ ▷ Set phase step
  $\Phi_{temp} \leftarrow \Phi_{opt}$ ▷ Set optimal phases
  for $j \leftarrow 1$ to n_{ch} **do**
    for $k \leftarrow 1$ to $2\pi/\varphi_{step}$ **do**
      flag $\leftarrow (-1)^i$ ▷ Alternate edge channels
      if flag=1 **then**
        $\Phi_{temp}[j] = \Phi_{temp}[j] + \varphi_{step}$
      else if flag=-1 **then**
        $\Phi_{temp}[-j] = \Phi_{temp}[-j] + \varphi_{step}$
      end if
      LOAD(Φ_{opt}) ▷ Load optimal phase settings
      READ(P_{temp}) ▷ Read power
      if $P_{temp} > P_{opt}$ **then**
        $\Phi_{opt} = \Phi_{temp}$ ▷ Set new optimal phases
        $P_{opt} = P_{temp}$ ▷ Set new optimal power
      end if
    end for
  end for
end for

After this step, the effective loss due to imperfect phase calibration is estimated with the imaging readout. Assuming minimal RF phase imbalance between channels throughout the imaging readout and RF readout, the effective phase calibration loss or efficiency (η_{cal}) is characterized as

$$\eta_{cal} = \frac{\left|\sum_n \tilde{v}_n\right|^2}{\left(\sum_n |\tilde{v}_n|\right)^2} \tag{S11}$$

where $\tilde{v}_n$ is the complex phasor for the classical 5 MHz signal of channel n. Since each channel's output voltage is digitized with the imaging readout, phase and amplitude information of each channel's 5 MHz classical signal can be extracted. Channel signals are filtered with a digital bandpass filter with a center frequency of 5 MHz and a bandwidth of 1 kHz. Hilbert transform is applied to each filtered channel signal to extract the amplitudes and are then summed, yielding the denominator, $\sum_n |\tilde{v}_n|$. The filtered channel

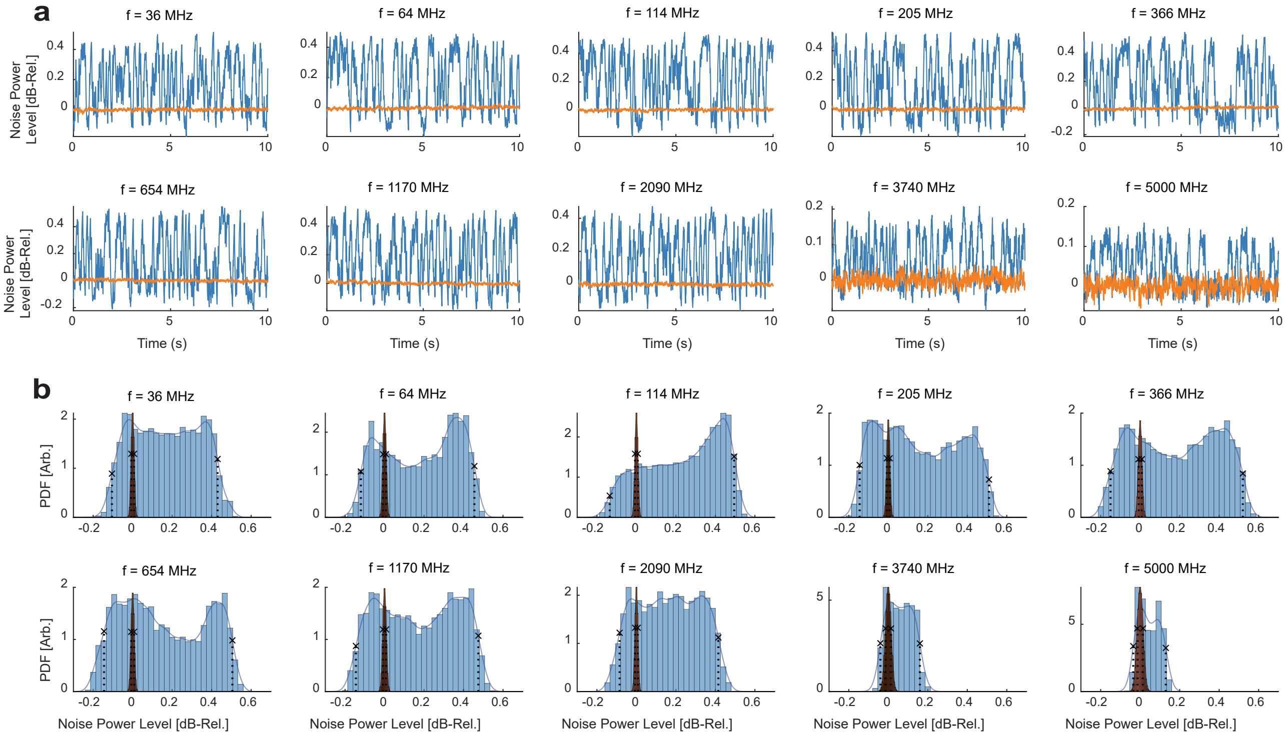

SUPPLEMENTARY FIG. S18. **High bandwidth measurement of squeezed light**. a) Noise power traces for the squeezed vacuum (blue) and vacuum (orange) states at different center frequencies. b) Histograms of the sampled noise powers for the squeezed vacuum (blue) and vacuum (orange) states. The KDE for the squeezed vacuum histogram is plotted in blue and a Gaussian fit to the vacuum histogram is plotted in black. The locations of the peak slopes for the squeezed state KDE (blue) and vacuum state Gaussian (black) are indicated with crosses and dashed lines, which yield the squeezing/antisqueezing level estimates and the noise floor.

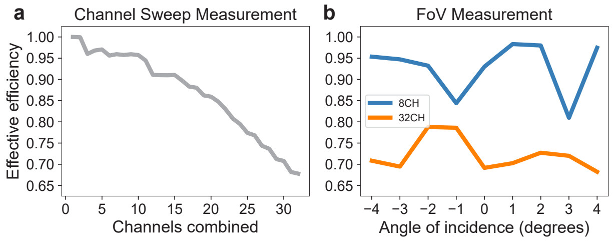

SUPPLEMENTARY FIG. S19. a) Effective phase calibration efficiencies (η_{cal}) for different channel combinations in the channel sweep measurement in Fig. 5b. b) Effective phase calibration efficiencies (η_{cal}) for different angles of incidence in the FoV measurement in Fig. 5f.

signals are also summed first and a Hilbert transform is applied to the summed signal to extract the amplitude, yielding the numerator, $\left|\sum_n \tilde{v}_n\right|$. The effective phase calibration efficiencies for different channel combinations of the channel sweep measurement in Fig. 5b are shown in Fig. S19a. In this measurement, effective phase calibration losses for 8 and 32 channels combined are 0.188 dB and 1.69 dB, respectively. The effective phase calibration loss of the power sweep measurement (32 channels combined) in Fig. 5c is 1.69 dB. The effective phase calibration losses of the beamwidth measurement in Fig. 5e for 8 and 32 channels combined are 0.0198 dB and 0.961 dB, respectively. The effective phase calibration efficiencies for different angles of incidence of the FoV measurement in Fig. 5f are shown in Fig. S19b. In this measurement, the minimum (maximum) phase calibration losses for 8 and 32 channels combined 0.0735 dB (0.918 dB) and 1.04 dB (1.66 dB), respectively.

VII. MEASUREMENT CHARACTERIZATION

A. Squeezed light imaging

1. *Data acquisition and analysis*

To image the squeezed light incident on the chip, we collect quadrature statistics of each antenna field mode over various phases by applying a 2π phase ramp on the LO at 0.5 Hz. The RF outputs from each QRX are digitized and stored at a sampling rate of 20 MSa/s over 4 seconds. A digital bandpass filter with a 2 MHz bandwidth is applied to the digital data. The data is then passed through a moving mean and variance filter with a bin size of 260,000 and is downsampled by a factor of 16,000 to obtain the sample means and variances.

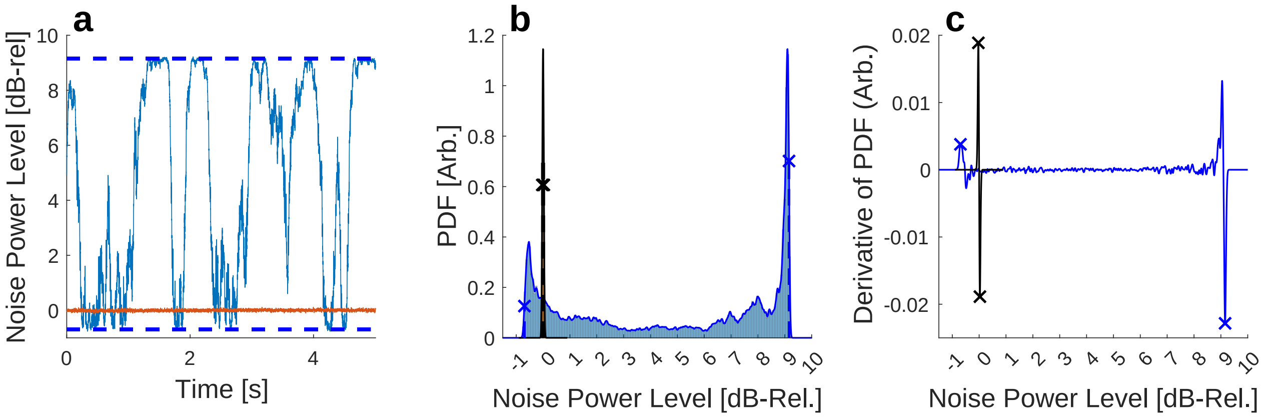

SUPPLEMENTARY FIG. S20. a) A five-second snippet of noise power levels for squeezed vacuum (blue) and vacuum (orange) measured at the source immediately before imaging. The noise powers were measured with a sampling rate of 20 kHz, center frequency of 6 MHz, 8 MHz RBW, and 100 Hz VBW. b) Histograms for the squeezed vacuum (light blue) and vacuum (orange) for the noise powers accumulated in a). The kernel density estimates (KDEs) for the squeezed vacuum (blue) and vacuum (black) histograms are plotted as solid lines. c) Derivative of KDEs for squeezed vacuum (blue) and vacuum (black).

2. *Squeezing parameter estimation*

The squeezing parameter was estimated with the source characterization setup of Fig. S17 in Configuration 1. Five-second traces of the squeezed vacuum and vacuum noise power levels immediately before transmission to the chip are shown in Fig. S20a. The corresponding histograms, kernel density estimates (KDEs), and derivatives of the KDEs are in S20b-c. The estimates for the squeezed state are indicated with the blue crosses. From the squeezing estimate of -0.695 ∓ 0.029 dB and antisqueezing estimate of 9.158 ± 0.029 dB, we obtain $r = 1.945^{-0.006}_{+0.015}$ and $\eta = 0.151^{+0.003}_{-0.006}$.

These numbers are within the error bars of the prediction from the source characterization of the SPDC waveguide (HCP SC18068). From the least-squares fit to the data in Fig. S17a, $\eta = 0.146$ and $\mu = 0.119$ $[\mathrm{mW}]^{-1/2}$. For the waveguide-coupled pump power of $P = 315.6 \pm 47.3$ mW, the estimated squeezing parameter is $r = 2.114 \pm 0.159$.

3. *Phase estimation*

The phases for the Wigner functions of the 32 antenna modes are found by performing a sinusoidal fit to a portion of the data with an approximately uniform phase ramp. The data and fits are shown in Fig. S22.

4. *Channel effective efficiency estimation*

The channel effective efficiencies are calculated from

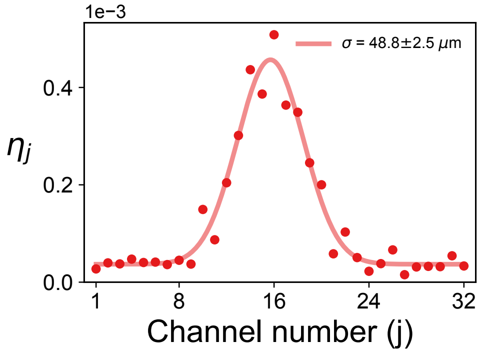

SUPPLEMENTARY FIG. S21. Channel effective efficiencies versus channel number. The solid line is a Gaussian fit to the data.

$$\eta = \frac{(A-1)\exp(2r)}{(\exp(2r)-1)(A+\exp(2r))}, \tag{S12}$$

where $A = \Delta Q_+^2/\Delta Q_-^2$ is the ratio of the antisqueezing (ΔQ_+^2) to squeezing (ΔQ_-^2) levels and $r = 1.945$. The ratio of antisqueezing to squeezing is obtained from the amplitudes of the sinusoidal fits in Fig. S22. The channel effective efficiencies are plotted in Fig. S21. For the Wigner functions, the geometric efficiency ($\eta_j^{(g)}$) for channel j is calculated as

$$\eta_j^{(g)} = \frac{\eta_j}{\sum_j \eta_j}, \tag{S13}$$

where $\sum_j \eta_j = 0.017$. A Gaussian fit to the data yields a standard deviation of $\sigma = 48.8 \pm 2.5$ μm. This corresponds to a Gaussian beam diameter of $4\sigma = 195.2 \pm 10$ μm, consistent with the collimator beam diameter of 200 μm.

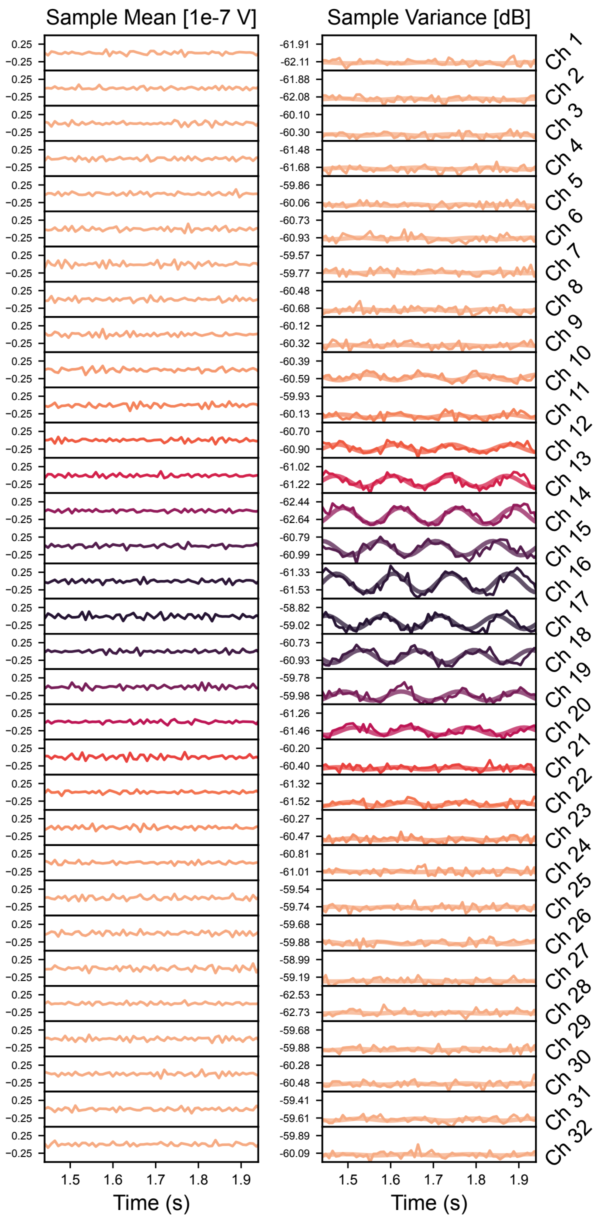

SUPPLEMENTARY FIG. S22. Quadrature sample means and variances over time for all 32 channels. The sample means are approximately zero, while sinusoidal variations are observed in the sample variances. The fits to the variances are plotted as transparent solid lines.

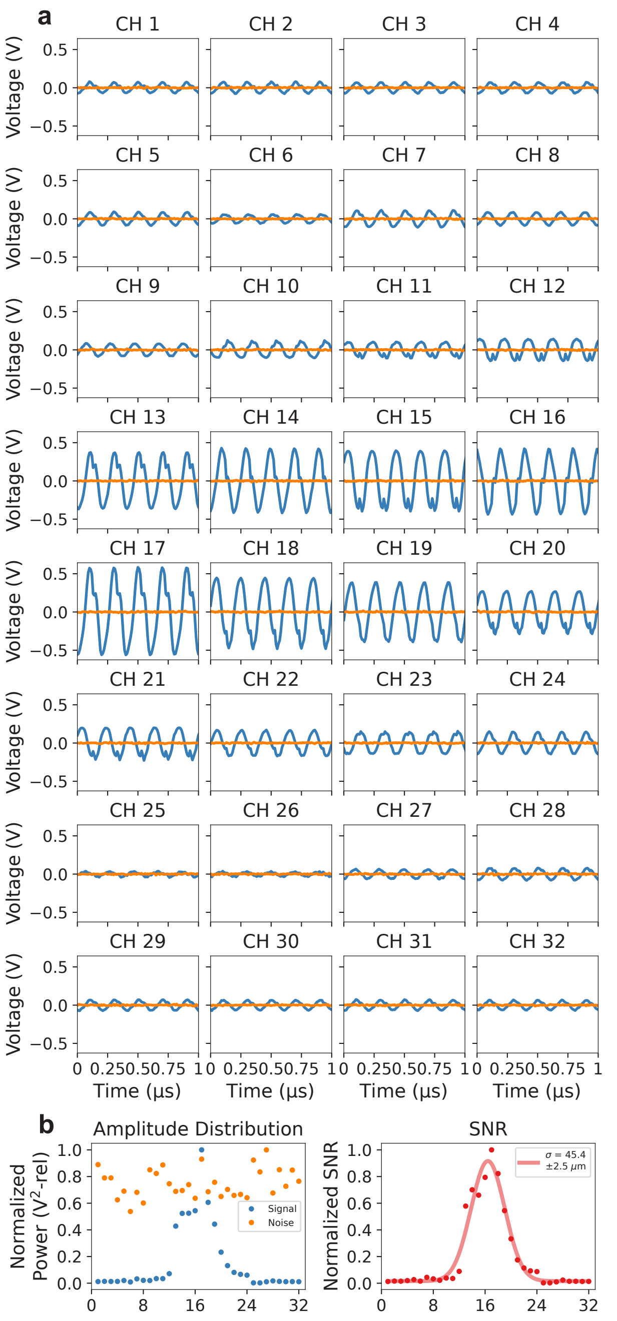

SUPPLEMENTARY FIG. S23. a) An example of signal (blue) and noise (orange) time-domain traces recorded for 32 channels. b) Extracted signal and noise powers across 32 channels in the frequency range of the downconverted tone and the corresponding SNR.

5. *Classical imaging*

Replacing the transmitted squeezed vacuum state with a coherent state allows operating the chip in a classical mode, compatible with previously demonstrated classical imaging schemes [18]. This enables hybrid quantum-classical operation of the chip and allows for comparing squeezed light measurements with classical measurements. In the case of imaging, we send a 1550 nm coherent state to the aperture through the collimator and apply 5 MHz phase modulation to the LO. The classical SNR is the same as effective efficiency (η), characterizing the decoherence of the quantum state in the receiver chain. Therefore, we approximate the classical SNR of this 5 MHz downconverted signal as a classical comparison to the squeezed light data. 32-channel RF outputs are digitized by a 32-channel digitizer with a sampling rate of 100 MSa/s. 10 ms of data is collected for each channel simultaneously using the same electronic readout as the squeezed light imaging measurement. By recording the outputs with and without sending light to the aperture, signal, and noise traces are collected for all

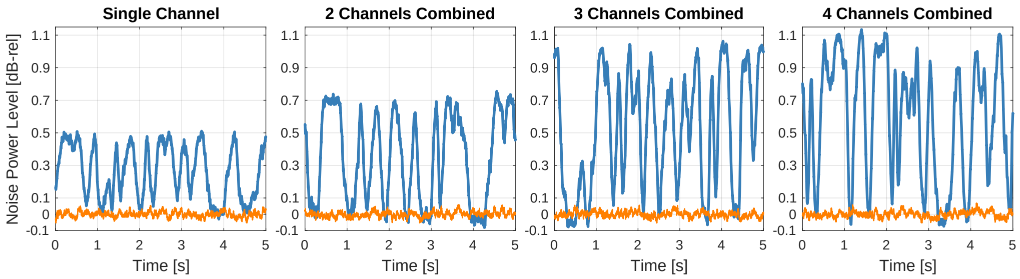

SUPPLEMENTARY FIG. S24. Beamforming on-chip with the HCP SC18068 waveguide for SPDC.

32 channels as seen in Fig. S23a. The raw traces are filtered with a digital bandpass filter with a center frequency of 5 MHz and a bandwidth of 100 kHz. Hilbert transform is taken of the filtered signal data to extract the analytical signal and the amplitude for each signal trace is determined as seen in Fig. S23b. Similarly, variances of the filtered noise data are measured and plotted in Fig. S23b. The ratio between these amplitudes gives the SNR, which is plotted in Fig. S23b. A Gaussian fit is applied to the SNR data with a standard deviation of $\sigma = 45.4 \pm 2.5$ μm.

B. Beamforming and pump power sweep

The beamforming (Fig. 5b) and 32-channel source characterization (Fig. 5c) data of the main text were performed with the source in Configuration 2, using the Covesion H-spec waveguide for SPDC. The beamforming sweep was performed at a waveguide-coupled pump power of $P = 383.3 \pm 19.2$ mW. From the Covesion H-spec characterization in Sec. IV, this corresponds to a squeezing parameter of $r = 0.748 \pm 0.019$.

Before performing the full 32-channel sweep, we first performed beamforming up to four channels using the HCP SC18068 waveguide for SPDC. A pump power of 226.8 ± 22.7 mW was coupled onto the waveguide, corresponding to a squeezing parameter of $r = 1.792 \pm 0.090$. The data are shown in Fig. S24.

1. *Classical channel sweep*

The classical SNR data to extract the estimated efficiencies for the channel sweep (Fig. 5b) of the main text is done by using the same electronic readout with the ESA. We send a 1550 nm coherent state to the aperture through the collimator and apply 5 MHz phase modulation to the LO. We measure the downconverted 5 MHz signal in the ESA for each channel combination ([1,2,4,6,8,10,12,14,16,18,20,22,24,26,28,30,32] channels) as seen in Fig. S25a. The video bandwidth for the signal measurements is 1 Hz, and the resolution bandwidth is 100 Hz. We also measure the noise powers for each channel combination using the ESA in zero-span mode at a center frequency of 5 MHz, with a resolution bandwidth of 2 MHz and a video bandwidth of 5 Hz as seen in Fig. S25a. After collecting these traces, we measure the signal and noise amplitudes as seen in Fig. S25b. For signal, we measure the total power in the frequency range between 4.95 MHz to 5.05 MHz in the collected data. For noise, we measure the mean power in the noise floor. We then extract the normalized SNR by taking the ratio of these amplitudes and normalizing the values as seen in Fig. S25b. The resulting values are used to plot the expected squeezing and antisqueezing using Eq. S8 with the squeezing parameter extracted from source characterization. For the proportionality constant, we apply a least-squares fit with η as the floating parameter with the squeezing parameter bounded as $r = 0.748 \pm 0.019$ to fit the resulting plot to the data, yielding a proportionality constant of $\eta_c = 0.021$.

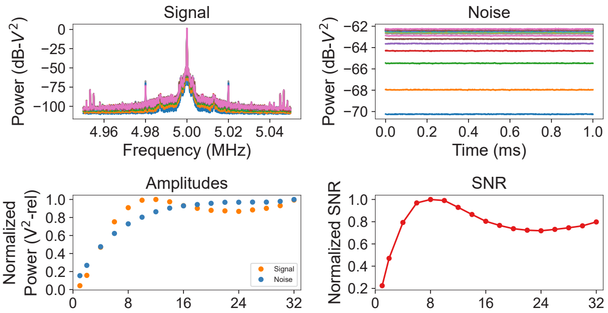

SUPPLEMENTARY FIG. S25. a) Signal and noise data recorded in the frequency domain for [1,2:2:32] channel combinations for the measurement in Fig. 5b. b) Extracted signal and noise powers in the frequency range of the downconverted tone and the corresponding SNR.

C. Beamwidth

The beamwidth measurement (Fig. 5e) of the main text was performed with the source in Configuration 4, using the Covesion M-spec waveguide for SPDC. The beamwidth data were taken at a waveguide-coupled pump power of $P = 383.6 \pm 19.7$ mW. From the Covesion M-spec characterization in Sec. IV, this corresponds to a squeezing parameter of

$r = 0.607 \pm 0.015$.

1. *Classical beamwidth*

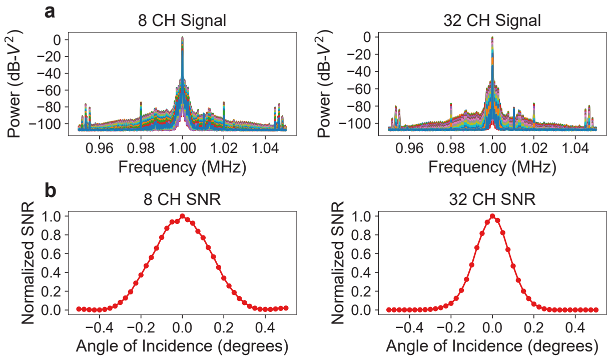

SUPPLEMENTARY FIG. S26. a) Signal data recorded in the frequency domain for different angles of incidence for 8 and 32 channels combined for the measurement in Fig. 5e. b) Extracted signal powers corresponding to normalized SNR in the frequency range of the downconverted tone for 8 and 32 channels combined.

The classical SNR data to extract the estimated efficiencies for the beamwidth measurements (Fig. 5e) of the main text is done by using the same electronic readout with the ESA. We send a 1550 nm coherent state to the aperture through the collimator and apply 1 MHz phase modulation to the LO. We measure the downconverted 1 MHz signal in the ESA for each incidence angle for both 8 channels and 32 channels combined, as seen in Fig. S26a. The video bandwidth for the signal measurements is 1 Hz, and the resolution bandwidth is 100 Hz. Since the electronic configuration for these measurements stays the same, the noise floor doesn't change, making signal directly proportional to SNR. Using the measured spectra in Fig. S26a, we measure the total power in the frequency range between 0.95 MHz to 1.05 MHz to acquire the signal amplitudes proportional to SNR for both 8 channels and 32 channels combined, as seen in Fig. S26b. The resulting values are used to plot the expected squeezing and antisqueezing using Eq. S8 with the squeezing parameter extracted from source characterization. For the proportionality constant, we apply a least-squares fit with η as the floating parameter and the squeezing parameter bounded as $r = 0.607 \pm 0.0152$ to fit the resulting plot to the data, yielding an optimal squeezing parameter of $r = 0.611$ and proportionality constants of $\eta_c^{(8)} = 0.0191$ and $\eta_c^{(32)} = 0.0141$ for 8 and 32 channels combined, respectively.

D. Field of view

The field of view (FoV) experiment (Fig. 5f) of the main text was performed with the source in Configuration 3, using the HCP SC19075 waveguide for SPDC. The FoV data were taken at a waveguide-coupled pump power of $P = 153.4 \pm 15.3$ mW. From the HCP SC19075 characterization in Sec. IV, this corresponds to a squeezing parameter of $r = 0.865 \pm 0.043$.

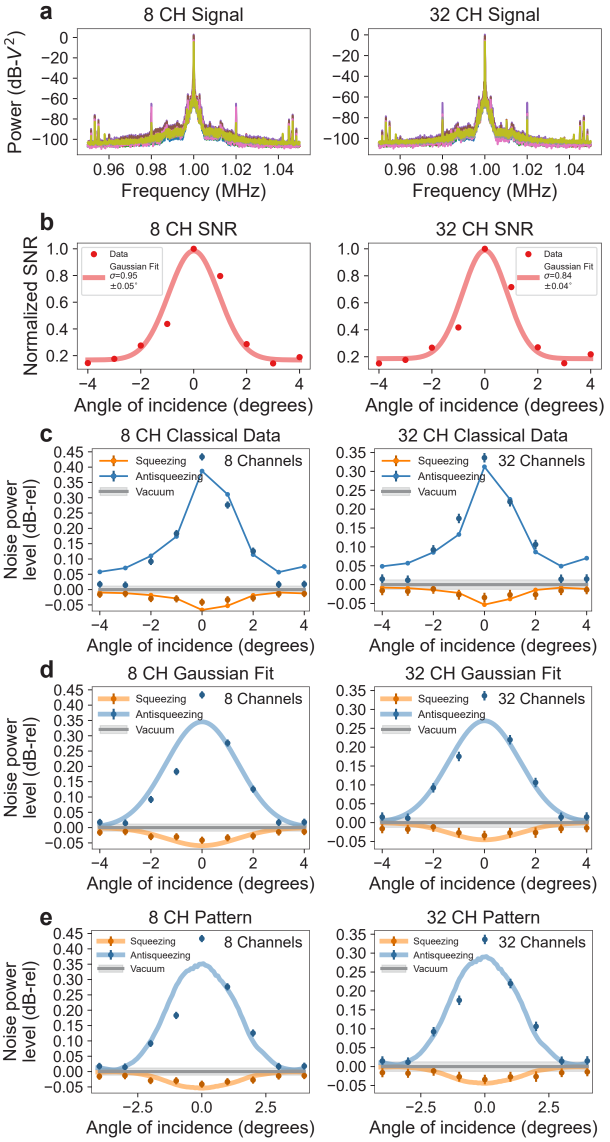

SUPPLEMENTARY FIG. S27. a) Signal data recorded in the frequency domain for different angles of incidence for 8 and 32 channels combined for the measurement in Fig. 5f. b) Extracted signal powers corresponding to normalized SNR in the frequency range of the downconverted tone for 8 and 32 channels combined. A Gaussian fit is applied to the data, yielding $\sigma = 0.95 \pm 0.05°$ and $\sigma = 0.84 \pm 0.04°$ for 8 and 32 channels combined, respectively. c) Comparison of the classical data to the squeezed light data. d) Comparison of the Gaussian fit of the classical data to the squeezed light data. e) Comparison of single antenna radiation pattern to the squeezed light data repeated from Fig. 5f.

1. Classical field of view

Multiple approaches were taken to acquire the classical comparison for the FoV measurement (Fig. 5f) of the main text. The single antenna radiation pattern is an accurate classical estimate of the full array pattern since the aperture is fully filled, and the electronic configuration stays the same for each angle of incidence. Therefore, the classical comparison in Fig. 5f uses the measured far-field single antenna radiation pattern shown in Fig. S6. We normalize this pattern and set it to be proportional to SNR. The resulting values are used to plot the expected squeezing and antisqueezing using Eq. S8 with the squeezing parameter extracted from source characterization, as shown in Fig. 5f and also in Fig. S27e. For the proportionality constant, we apply a least-squares fit with η as the floating parameter and with the squeezing parameter bounded as $r = 0.865 \pm 0.043$ to fit the resulting plot to the data, yielding an optimal squeezing parameter of $r = 0.908$ and proportionality constants of $\eta_c^{(8)} = 0.0167$ and $\eta_c^{(32)} = 0.0152$ for 8 and 32 channels combined, respectively.

A beamformed classical measurement is also taken for each of the 9 angles in the FoV measurement. For classical FoV measurement, we send a 1550 nm coherent state to the aperture through the collimator and apply 1 MHz phase modulation to the LO. We measure the downconverted 1 MHz signal in the ESA for each incidence angle for both 8 channels and 32 channels combined, as seen in Fig. S27a. The video bandwidth for the signal measurements is 1 Hz, and the resolution bandwidth is 100 Hz. Since the electronic configuration for these measurements stays the same, the noise floor doesn't change, making signal directly proportional to SNR. Using the measured spectra in Fig. S27a, we measure the total power in the frequency range between 0.95 MHz to 1.05 MHz to acquire the signal amplitudes proportional to SNR for both 8 channels and 32 channels combined as seen in Fig. S27b. The resulting values are used to plot the expected squeezing and antisqueezing using Eq. S8 with the squeezing parameter extracted from source characterization. For the proportionality constant, we apply a least-squares fit with η as the floating parameter and with the squeezing parameter bounded as $r = 0.865 \pm 0.043$ to fit the resulting plot to the data, yielding an optimal squeezing parameter of $r = 0.908$ and a proportionality constant of $\eta_c^{(8)} = 0.0181$ and $\eta_c^{(32)} = 0.0145$ for 8 and 32 channels combined, respectively. The comparison of the classical data to the squeezed light data in Fig. 5f of main text is shown in Fig. S27c. We also fit the classical data to a Gaussian function, yielding a standard deviation of 0.95±0.05 degrees and 0.84±0.04 degrees for 8 and 32 channels combined, respectively. The comparison of the Gaussian fit to the squeezed light data is shown in Fig. S27d.

A linear interpolation is used to extract the classical FoV to directly compare with the FoV extracted from squeezed light data in the main text. With linear interpolation on the classical data, we extract an FoV of 2.5 degrees and 2.3 degrees for 8 and 32 channels combined, respectively. These classical estimates match well with the squeezed light FoV of 2.3 degrees and 2.7 degrees for 8 and 32 channels combined, respectively, in the main text. The discrepancy between classical and squeezed light data is due to the measurements being taken sequentially. Future schemes in which classical and non-classical light are multiplexed in the same link [19] would minimize this discrepancy.

VIII. LOSS BUDGET AND IMPROVEMENTS

A. System loss budget

The components in the phased array system were optimized to minimize signal loss as much as possible. The system losses can be broken down into source loss, free-space loss, on-chip loss, and RF loss.

1. Source loss

Losses of the fiber-optic components in the source subsystem are 0.8 dB loss from the collimator, <0.5 dB loss from the polarization controller, 0.55 dB loss from the isolator, 0.15 dB loss from the optical switch, and 1 dB loss from the fiber-optic cables and connectors. Losses for each SPDC waveguide configuration are in Sec. IV.

2. Free-space loss

Free-space loss consists of the scattering loss from free-space propagation and geometric loss of the incident beam and the aperture. Due to the relatively short free-space link distance (<1 m) in the proof-of-concept demonstrations, free-space propagation loss is negligible. The geometric loss is characterized by calculating the modal overlap between the incident free-space beam mode and the on-chip aperture mode. For the weighted 32-channel aperture with a 200 μm collimated beam, the geometric loss is 1.14 dB.

3. On-chip loss

On-chip losses are mentioned in Methods but will be repeated for completeness. They consist of 3.78 dB loss from simulated antenna insertion loss, 0.321 dB loss from waveguide propagation, and 1.52 dB loss from photodiode quantum efficiency. This results in a total expected on-chip loss of 5.62 dB. The on-chip losses are verified experimentally by sending 200 μm collimated beam to the chip aperture after setting all QRXs to the unbalanced (100:0) configuration and summing all QRX currents. For 0.452 mW input

power, the output current is 0.0615 μA, resulting in an insertion loss of 8.66 dB. For a 200 μm collimated beam, the geometric loss is 1.14 dB, the insertion loss of the collimator is 0.8 dB, and the connector loss is expected to be <1 dB. De-embedding these losses from the measurement, the on-chip losses are measured to be 5.72 dB, which agrees within 0.1 dB of the expected 5.62 dB loss.

4. RF loss

RF loss sets the QRX shot noise clearance for a given hardware chain, accounting for the noise of the electronic components. For the imaging measurement in Fig. 4, across the bandwidth of the measurement, the RF loss due to the shot noise clearance has a median of 0.128 dB and a mean of 0.137 dB. For the squeezed light measurements in Fig. 5, the RF loss due to the shot noise clearance for 8 channels and 32 channels combined is 0.287 dB and 0.472 dB, respectively. In addition to shot noise clearance, when different channels are combined in RF, phase errors during phase calibration will cause decoherence to the received quantum state, leading to additional loss. This loss was characterized classically using the 32-channel readout as detailed in Section VI.

B. Measurement loss budget

We characterize the measurement setup losses for each experiment and report them here. A summary of the system losses for each measurement setup can be seen in Table S3.

Experiment	Source loss (dB)	Free-space loss (dB)	On-chip loss (dB)	RF loss (dB)	Total loss (dB)
Imaging	8.17	1.14	5.72	0.137	15.2
Channel sweep	6.97	2.18	5.72	0.660	15.5
Power sweep	6.97	4.85	5.72	2.16	19.7
BW (8CH)	6.97	2.18	5.72	0.306	15.2
BW (32CH)	6.97	4.85	5.72	1.43	19.0
FoV (8CH)	6.90	2.18	5.72	1.21	16.1
FoV (32CH)	6.90	4.85	5.72	2.13	19.6

SUPPLEMENTARY TABLE S3. Measurement setup system losses for each experiment

1. Squeezed light imaging

The total expected efficiency for the imaging measurement in Fig. 4 from the characterized component losses outlined in Sec. VIII A is 0.0304, corresponding to 15.2 dB loss. Source loss, free-space loss, on-chip loss, and RF loss account for 8.17 dB, 1.14 dB, 5.72 dB, and 0.137 dB, respectively.

2. Wavefunction engineering

a. Channel sweep: The total expected efficiency for the channel sweep measurement in Fig. 5b from the characterized component losses outlined in Sec. VIII A for 8 channels combined is 0.0280, corresponding to 15.5 dB loss. Source loss, free-space loss, on-chip loss, and RF loss account for 6.97 dB, 2.18 dB, 5.72 dB, and 0.660 dB, respectively.

b. Power sweep: The total expected efficiency for the power sweep measurement in Fig. 5c from the characterized component losses outlined in Sec. VIII A is 0.0107, corresponding to 19.7 dB loss. Source loss, free-space loss, on-chip loss, and RF loss account for 6.97 dB, 4.85 dB, 5.72 dB, and 2.16 dB, respectively.

c. Beamwidth: The total expected efficiencies for the beamwidth (BW) measurement in Fig. 5e from the characterized component losses outlined in Sec. VIII A are 0.0304 and 0.0127, corresponding to 15.2 dB and 19.0 dB loss, for 8 and 32 channels combined, respectively. For 8 channels combined, source loss, free-space loss, on-chip loss, and RF loss account for 6.97 dB, 2.18 dB, 5.72 dB, and 0.306 dB, respectively. For 32 channels combined, source loss, free-space loss, on-chip loss, and RF loss account for 6.97 dB, 4.85 dB, 5.72 dB, and 1.43 dB, respectively.

d. Field of view: The total expected efficiencies for the field of view (FoV) measurement in Fig. 5f from the characterized component losses outlined in Sec. VIII A are 0.0251 and 0.0110, corresponding to 16.1 dB and 19.6 dB loss, for 8 and 32 channels combined, respectively. For 8 channels combined, source loss, free-space loss, on-chip loss, and RF loss account for 6.90 dB, 2.18 dB, 5.72 dB, and 1.21 dB, respectively. For 32 channels combined, source loss, free-space loss, on-chip loss, and RF loss account for 6.90 dB, 4.85 dB, 5.72 dB, and 2.13 dB, respectively.

C. Loss improvements

System loss can be significantly improved by implementing phased arrays with low-loss on-chip components and sources already demonstrated in the literature as will be detailed below. To summarize, squeezed light sources with 0.042 dB loss [14], integrated photodiodes with 0.220 dB loss [20], nanophotonic antennas with 0.362 dB insertion loss [21, 22], and on-chip waveguides with <1 dB/m loss [23] have been demonstrated. Combining these components with the phased array concept allows us to reach sub-dB (down to 0.626 dB) system losses. Improvements in PIC fabrication can also further improve these losses in the future. Squeezed light sources with optical pre-amplification [24, 25] can allow a loss-tolerant approach to using squeezed light with our concept. Finally, while loss is more detrimental to continuous-variable quantum information, our quantum phased array concept can also be implemented to

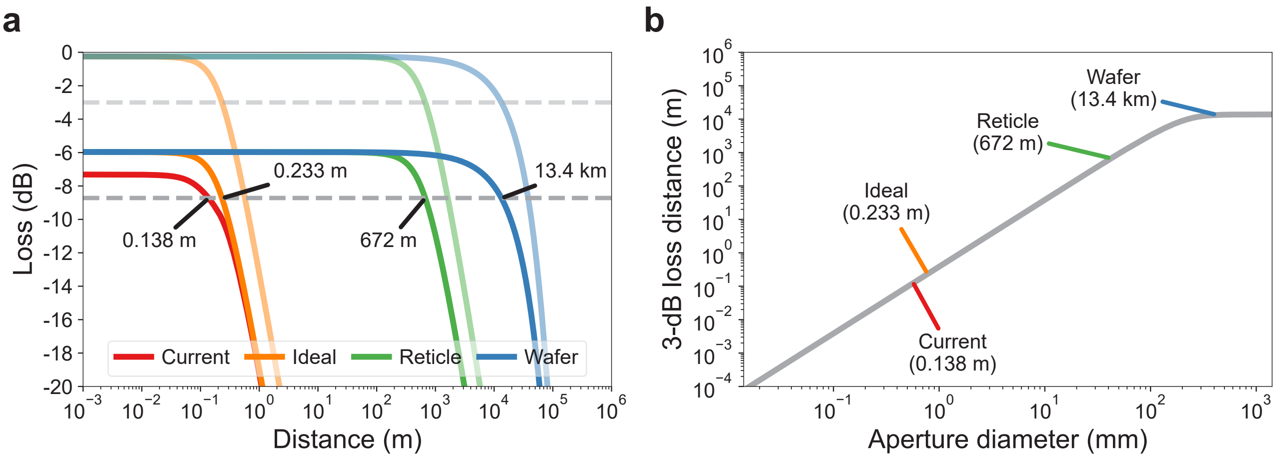

SUPPLEMENTARY FIG. S28. a) Change in total loss (solid lines) and free-space loss (faint lines) with varying TX-RX link distances for different apertures. Gray dashed line represents the 3-dB free-space loss distance. b) Distance needed to achieve 3-dB free-space loss with varying aperture diameters.

interface with discrete-variable quantum information using photon counting devices as receivers.

Therefore, these advances chart a clear path toward sub-dB system losses with a potential to enable sub-shot-noise level noise floors with 8.75 dB measured squeezing. On-chip integration of photonic and electronic components at scale [26, 27] also enables numerous opportunities to utilize non-classical resources in integrated photonic-electronic systems.

a. Source loss: Sources with 0.0420 dB loss have been demonstrated [14] in the literature that can reduce the source losses in our system. Integrated nonlinear photonic systems also offer further miniaturization of our approach by enabling transmitter counterparts of the demonstrated receiver. Squeezed light sources on thin-film lithium niobate [28] or silicon nitride [29, 30] can enable novel optoelectronic system architectures using this approach. Sources with preamplification using optical parametric amplifiers [24, 25] also offer a promising route to propel this approach toward practical applications.

b. Free-space loss: Using our approach, geometric loss can become negligible by deploying large active-area apertures with a large number of elements for sub-wavelength control of an incident field. Large-scale optical phased arrays for classical links have been demonstrated [13, 31] albeit with higher geometric losses. Scaling our design to a large number of channels can enable wireless quantum links with negligible loss due to precise mode matching from on-chip sub-wavelength control of non-classical light. A scaling analysis for this is done in Section IX.

c. On-chip loss: On-chip loss can be significantly reduced by using photodiodes with high quantum efficiency and improving the antenna design. Ge photodiodes with near-unity quantum efficiency can be fabricated on silicon photonics with 95% quantum efficiency (0.223 dB loss) photodiodes demonstrated in the literature [20] and >90% quantum efficiency (0.458 dB) photodiodes being available in many silicon photonic process design kits [32, 33]. Using multiple layers, insertion loss of the MMA can be reduced to be limited by the waveguide propagation loss with multi-layer grating couplers having been demonstrated with >92% coupling efficiency (0.362 dB loss) [21, 22]. Waveguide propagation loss on chip can be reduced from 2 dB/cm with silicon waveguides to < 1 dB/m with silicon nitride waveguides, reducing our waveguide propagation loss from 0.321 dB to < 0.002 dB [23, 34]. By applying the design methodology outlined in our work to multi-layer grating couplers and deploying low-loss waveguides and high quantum efficiency photodiodes, on-chip losses can be reduced to sub-dB levels.

d. RF loss: RF loss is limited by the SNC and CMRR of the QRXs, which corresponds to 0.00405 dB loss for high SNC QRX and 0.176 dB for high BW QRX. In the phased array system, high SNC QRXs are used. Therefore, RF loss in our system is negligible. Nevertheless, this RF loss can be further reduced by deploying photodiodes with high power handling and high linearity [35, 36] and maximizing LO power.

IX. SCALING ANALYSIS AND APPLICATIONS

Here, we model the loss of phased arrays for different aperture sizes and show how phased arrays can be used to mitigate geometric loss for long distance quantum links over free space. Since our design is modular, each channel in the system can be duplicated in design keeping the same architecture to scale to larger apertures. We exemplify four aperture sizes: 1) Current chip aperture characterized in Section I A. 2) Ideal chip aperture allowing perfect mode matching over $550 \times 550\ \mu m^2$. 3) Reticle-scale chip aperture allowing perfect mode matching over $30 \times 30\ mm^2$. 4) Wafer-scale chip aperture allowing perfect mode matching over $300 \times 300\ mm^2$. We name these four

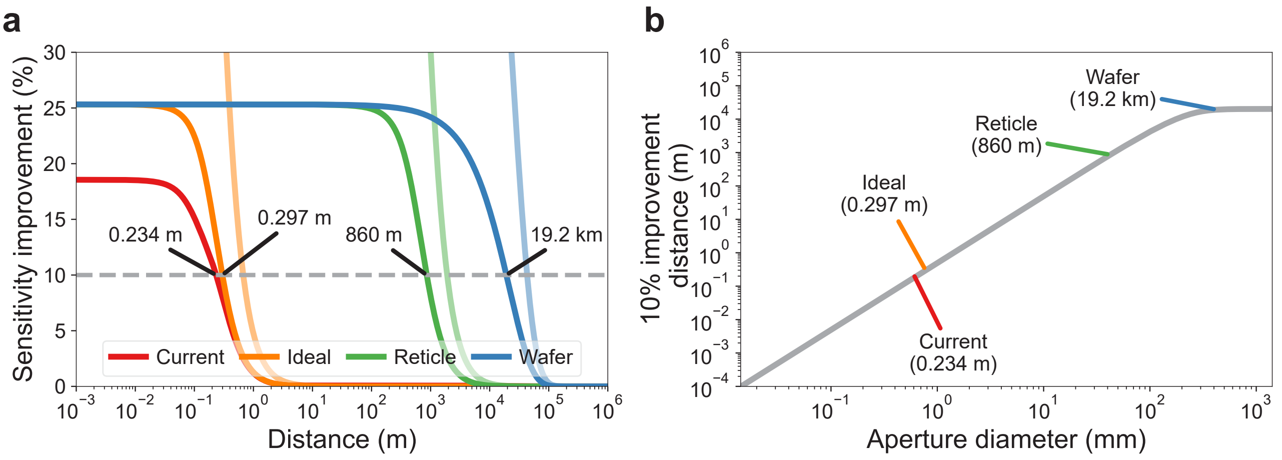

SUPPLEMENTARY FIG. S29. a) Sensitivity improvement below the shot noise floor with varying TX-RX link distance for phased array sensors with different aperture diameters. Solid lines correspond to the sensitivity improvement with added on-chip loss (5.72 dB) and faint lines correspond to sensitivity improvement without any loss except geometric loss. As seen from the red trace, up to 18% sensitivity improvement is possible with current geometric and on-chip losses. Dashed line represents the 10% sensitivity improvement distance. b) Distance needed to achieve 10% sensitivity improvement below the shot noise floor with varying aperture diameters.

scenarios as 1) Current, 2) Ideal, 3) Reticle, 4) Wafer, respectively.

For current and ideal, we assume a transmitter transmitting a Gaussian beam with 200 μm beam diameter, the same transmitter used in the main text experiments. For reticle and wafer, we assume a phased array transmitter that can generate a Gaussian beam with 15 mm and 150 mm beam diameters, respectively assuming a case in which reticle and wafer-sized phased array transmitters are used.

A. Loss analysis

Propagation of a collimated Gaussian beam with an initial beam diameter, w_0, expands the beam diameter with propagation distance, d, to result in a Gaussian beam with beam diameter, w.

$$w(d) = w_0\sqrt{1+\left(\frac{\lambda d}{\pi w_0^2}\right)^2} \tag{S14}$$

where λ is the wavelength for the monochromatic light. We model the propagation of the Gaussian beam transmitted from the collimator or phased array transmitter with this equation. In reality, the transmitted Gaussian beam would be truncated at the edges due to the finite aperture size of the transmitter. However, since we define a transmitted beam diameter half the transmitter aperture diameter, the effects of this truncation are assumed to be negligible. After the transmitted beam expands and hits the receiver aperture, we can derive the modal overlap using Eq. S1. The geometric efficiency, or mode matching efficiency, is then

$$\eta = \left[\text{erf}\left(\frac{a}{w\sqrt{2}}\right)\right]^2 \tag{S15}$$

where a is the side length of the receiver aperture and w is the beam diameter of the incident beam.

We also add atmospheric loss to geometric loss to obtain free-space loss. At 1550 nm, atmospheric attenuation can be assumed to be 0.2 dB/km, giving $\eta_a = 0.955$ [37]. Then,

$$\eta = (\eta_a)^{d/1000}\left[\text{erf}\left(\frac{a}{w\sqrt{2}}\right)\right]^2 \tag{S16}$$

Using this, we plot the loss vs. TX-RX link distance in Fig. S28a. Solid lines represent the model with added receiver on-chip loss (5.72 dB) and faint lines represent the model with only free-space loss. Intersection points between the solid lines and the dashed line give the link distance when there is 3-dB free-space loss. With this, we also plot the TX-RX link distance vs. aperture diameter to achieve 3-dB free-space loss in Fig. S28b. As seen in these plots, 3-dB loss is achieved at a link distance of 0.138 m for the current chip, 0.233 m for the ideal chip, 672 m for the reticle-scale aperture, and 13.4 km for the wafer-scale aperture. The saturation in distance at larger apertures is caused by the atmospheric loss and is not a limitation of the aperture. Without atmospheric loss (0.2 dB/km), the distances for reticle-scale and wafer-scale apertures could be pushed to 693 m and 69.3 km, respectively.

B. Quantum-enhanced sensing

With the aforementioned loss model, we connect the calculated losses to the maximum squeezing that can be measured over a free-space channel. Using squeezed light for quantum-enhanced sensing could entail a scheme, in which a highly transmissive or reflective sample is placed between a transmitter and a

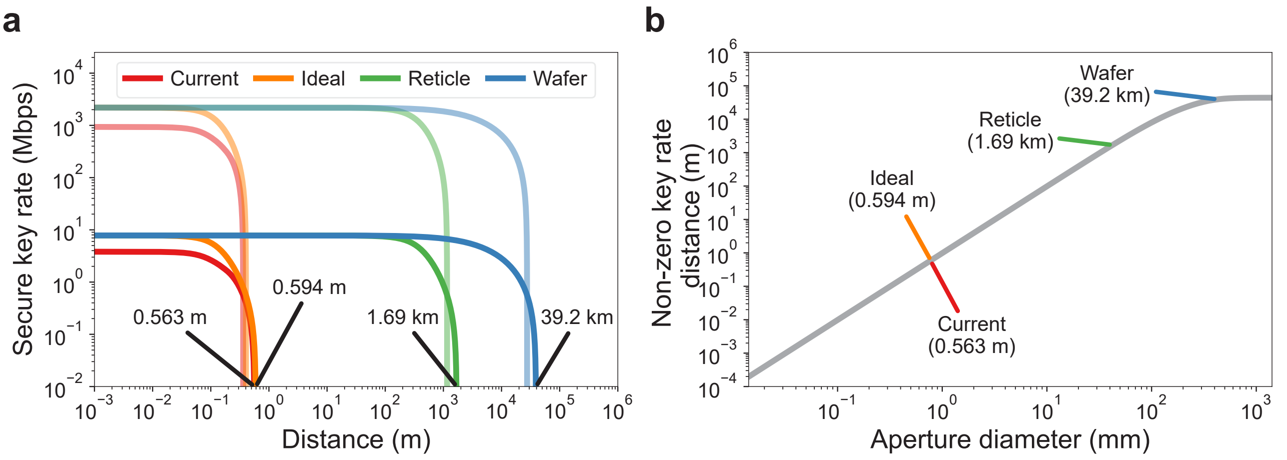

SUPPLEMENTARY FIG. S30. a) Secure key rate with varying TX-RX link distance for phased array transceivers with different aperture diameters. Solid lines represent a phased array system with high SNC QRX (30.3 dB SNC, 90.2 dB CMRR, 10.4 MHz BW_{3dB}), and faint lines represent a phased array system with high BW QRX (14.0 dB SNC, 90.2 dB CMRR, 2.57 GHz BW_{3dB}). All lines include the current on-chip losses (5.72 dB). b) Distance needed to achieve a non-zero secure key rate with varying aperture diameters for a phased array system with high SNC QRX.

receiver in the free-space channel. Squeezed light can be used to increase SNR for higher resolution or sensitivity [38]. There have been previous demonstrations of quantum-enhanced sensing with squeezed light for gravitational wave detection [39], microscopy [40, 41], optical phase estimation [28, 42], magnetic field sensing [43, 44], and LIDAR [45, 46]. These demonstrations can be deployed more broadly and practically with phased array sensors due to the precise mode matching achievable with sub-wavelength-engineered on-chip apertures. Assuming negligible loss from the sample and phase locking to the squeezed quadrature, measured squeezing estimates the sensitivity improvement a phased array sensor would enable below the shot noise limit, surpassing the standard quantum limit.

Using the loss estimate from before, we plot the sensitivity improvement vs. TX-RX link distance in Fig. S29a. Solid lines represent the model with added receiver chip losses (5.72 dB) and faint lines represent the model with only free-space loss, which approaches infinity in the limit of no loss and infinite squeezing. As seen from the red trace, up to 18% sensitivity improvement is possible with current free-space and on-chip losses. With the aforementioned improvements in component losses in Section VIII C achieving down to 0.626 dB system loss corresponding to 87% sensitivity improvement is possible. We also plot the TX-RX link distance vs. aperture diameter to achieve 10% sensitivity improvement in Fig. S29b. As seen in these plots, 10% sensitivity improvement is achieved at a link distance of 0.234 m for the current aperture, 0.297 m for the ideal aperture, 860 m for the reticle-scale aperture, and 19.2 km for the wafer-scale aperture, potentially enabling practical quantum enhancement in m-to-km range LiDAR [45, 46]. These estimates show that on-chip phased arrays can start to enable free-space losses low enough to deploy previously demonstrated squeezed light sensing schemes for practical applications. The saturation in distance at larger apertures is caused by the atmospheric loss and is not a limitation of the aperture. Without atmospheric loss (0.2 dB/km), the distances for reticle-scale and wafer-scale apertures could be pushed to 884 m and 88.4 km, respectively.

C. Quantum key distribution

Phased array transceivers could be used to achieve wireless quantum communication networks with each phased array acting as a node in the network [47–51]. Phased arrays could also be employed as relays at intermediate locations between nodes in a quantum network to increase the range of link distances. These wireless quantum networks could enable the deployment of quantum technologies in environments where it is impractical to build infrastructure or where mobility and reconfigurability is needed, potentially similar to the last-mile solution conventional wireless communications serve in classical communication networks. Phased array transceiver networks could also be scaled to realize large-scale hybrid wired and wireless quantum networks [47] that can implement atmospheric turbulence correction [52] and fast switching between nodes [53].

With the aforementioned loss model and the specifications of high-SNC QRX (30.3 dB SNC, 90.4 dB CMRR, 10.4 MHz BW_{3dB}, 381 MHz BW_{shot}) and high-BW QRX (14.0 dB SNC, 90.4 dB CMRR, 2.57 GHz BW_{3dB}, 3.70 GHz BW_{shot}), we connect the characterization results to performance in quantum communications, more specifically achievable secret key rates in Gaussian modulated continuous-variable quantum key distribution (CV QKD). In this scheme, a phased array transmitter can beamform in the di-

rection of a phased array receiver over free space and transmit displaced coherent states or squeezed states whose quadratures are modulated by a continuous Gaussian random variable, $\mathcal{N}(0, \tilde{V}_{mod})$. A phased array receiver would then beamform on the transmitted beam and acquire quadrature data. While this serves as an exemplary scheme, more complex protocols could also be realized by leveraging the multi-channel architecture of phased arrays.

For the Gaussian modulated CV QKD scheme, the secure key rate, K, is given by [54, 55]

$$K = B(fI_{AB} - \chi_{BE}) \tag{S17}$$

where B is the symbol rate, which we define to be the 3-dB bandwidth of the QRX ($\mathrm{BW_{3dB}}$) through which the reported SNCs are defined, f is the efficiency of the reconciliation algorithm, I_{AB} is the mutual information between the quadrature data at the two nodes, and χ_{BE} is the Holevo bound. Considering reverse reconciliation [56], the Holevo bound corresponds to the maximum accessible information that an eavesdropper could have on the receiver's data.

The mutual information I_{AB} can be computed from the Shannon entropy and the experimental parameters as follows

$$I_{AB} = \log_2(1 + \mathrm{SNR}) \tag{S18}$$

where the signal-to-noise ratio (SNR) is given by:

$$\mathrm{SNR} = \frac{\eta V_{\mathrm{mod}}}{1 + \xi_{tot}} \tag{S19}$$

where η is the optical loss between transmission and reception including the quantum efficiency of the receiver photodiodes, V_{mod} is the quadrature modulation variance related to the mean photon number of the transmitted state, $\langle n \rangle$, as $\langle n \rangle = \frac{1}{2}V_{mod}$. We assume a typical V_{mod} of 1. ξ_{tot} is the excess noise introduced by the optical loss and finite SNC and CMRR of the QRX [54, 55].

$$\xi_{tot} = \xi_{geom} + \xi_{chip} + \xi_{\mathrm{SNC}} + \xi_{\mathrm{CMRR}} + \xi_{atm} \tag{S20}$$

Assuming independent noise sources, ξ_{tot} is the sum of the noise variances referred to the transmitter in shot noise units (normalized to the shot noise level). ξ_{geom} is the noise introduced by the free-space loss, $\xi_{geom} = (1 - \eta_{geom})/\eta_{geom}$. ξ_{chip} is the noise introduced by the on-chip loss, $\xi_{chip} = (1 - \eta_{chip})/\eta_{chip}$. ξ_{SNC} is the noise introduced by the electronics that can be derived in terms of SNC using Eqs. (S3) and (S5), $\xi_{\mathrm{SNC}} = 1/(\mathrm{SNC}-1)$. ξ_{CMRR} is the noise introduced by the LO suppressed by the CMRR that can be derived using Eq. (S3), $\xi_{\mathrm{CMRR}} = \mathrm{RIN}P_{LO}/(4\mathrm{CMRR})$. We assume a shot-noise-limited LO (i.e. $\mathrm{RIN}P_{LO} = 1$). ξ_{atm} is the noise introduced by the atmospheric loss for 1550 nm light, which is 0.2 dB/km [37].

Lastly, the calculation of the Holevo bound requires the covariance matrix between the data at the two nodes, which depends on the experimental parameters. A detailed description for estimating the Holevo bound is provided in [54, 55]. Once the mutual information I_{AB} and the Holevo bound χ_{BE} are computed, K can be directly obtained from Eq. S17. The reconciliation efficiency f is assumed to be 0.95, as reported in [57].

We plot the secure key rate vs. TX-RX link distance in Fig. S30a. Solid lines represent a phased array system with high SNC QRX (30.3 dB SNC, 90.4 dB CMRR, 10.4 MHz bandwidth), and faint lines represent a phased array system with high BW QRX (14.0 dB SNC, 90.4 dB CMRR, 2.57 GHz bandwidth). All lines include the current on-chip losses (5.72 dB). From this plot, the secure key rate for the current phased array aperture can be up to 3.81 Mbps and the secure key rate for the ideal aperture can be up to 7.82 Mbps. If the PIC is packaged with a 32-channel high BW TIA array, this can be pushed up to 0.935 Gbps for the current aperture and up to 2.19 Gbps for the ideal aperture.

We also plot the TX-RX link distance vs. aperture diameter to achieve a non-zero secure key rate for a phased array system with high SNC QRX in Fig. S30b. The difference in the non-zero key rate distance between high SNC and high BW configurations comes from the different SNCs between the two configurations. As seen in this plot, the non-zero key rate distance is 0.563 m for the current aperture, 0.594 m for the ideal aperture, 1.69 km for the reticle-scale aperture, and 39.2 km for the wafer-scale aperture. The saturation in distance at larger apertures is caused by the atmospheric loss and is not a limitation of the aperture. Without atmospheric loss (0.2 dB/km), the distances for reticle-scale and wafer-scale apertures could be pushed to 1.77 km and 177 km, respectively.

These results are promising, showing that the current chip is capable of enabling chip-scale wireless QKD at m-scale distances. This can potentially enable QKD in environments where RFID chips are used, such as on credit cards, point-of-sale devices, ATMs, or in warehouses for product authentication. Projected ranges with reticle-scale and wafer-scale apertures are promising for long-range QKD, enabling long-distance quantum communications [58] with chip-scale devices. Due to the size, weight and power advantage of chip-scale systems, phased array transceiver chips could enable mobile quantum communication networks with drones [59], satellites [48], or portable devices, such as point-of-sale devices and credit cards.

[1] Zhang, Y. *et al.* A compact and low loss y-junction for submicron silicon waveguide. *Opt. Express* **21**, 1310–1316 (2013).

[2] Van Laere, F. *et al.* Compact and highly efficient grating couplers between optical fiber and nanophotonic waveguides. *Journal of Lightwave Technology* **25**, 151–156 (2007).

[3] Vermeulen, D. *et al.* High-efficiency fiber-to-chip grating couplers realized using an advanced cmos-compatible silicon-on-insulator platform. *Opt. Express* **18**, 18278–18283 (2010).

[4] Raval, M., Poulton, C. V. & Watts, M. R. Unidirectional waveguide grating antennas with uniform emission for optical phased arrays. *Opt. Lett.* **42**, 2563–2566 (2017).

[5] Vivien, L. & Pavesi, L. *Handbook of silicon photonics* (Taylor & Francis, 2016).

[6] Gurses, V. *et al.* A compact silicon photonic quantum coherent receiver with deterministic phase control. In *2023 Conference on Lasers and Electro-Optics (CLEO)*, 1–2 (2023).

[7] Gurses, B. V. & Hajimiri, A. Performance limits of sub-shot-noise-limited balanced detectors. In *Frontiers in Optics + Laser Science 2022 (FIO, LS)*, JW4A.32 (Optica Publishing Group, 2022).

[8] Bruynsteen, C., Vanhoecke, M., Bauwelinck, J. & Yin, X. Integrated balanced homodyne photonic–electronic detector for beyond 20 GHz shot-noise-limited measurements. *Optica* **8**, 1146–1152 (2021).

[9] Tasker, J. F. *et al.* Silicon photonics interfaced with integrated electronics for 9 GHz measurement of squeezed light. *Nature Photonics* **15**, 11–15 (2021).

[10] Gurses, V., Sarkar, D., Davis, S. & Hajimiri, A. An integrated photonic-electronic quantum coherent receiver for sub-shot-noise-limited optical links. In *Optical Fiber Communication Conference (OFC) 2024*, Tu2C.1 (Optica Publishing Group, 2024).

[11] Texas Instruments. *4.25-Gbps Transimpedance Amplifier With AGC and RSSI* (2011).

[12] Williams, K. J. & Esman, R. D. Design considerations for high-current photodetectors. *J. Lightwave Technol.* **17**, 1443 (1999).

[13] Chung, S., Abediasl, H. & Hashemi, H. A monolithically integrated large-scale optical phased array in silicon-on-insulator cmos. *IEEE Journal of Solid-State Circuits* **53**, 275–296 (2018).

[14] Vahlbruch, H., Mehmet, M., Danzmann, K. & Schnabel, R. Detection of 15 dB squeezed states of light and their application for the absolute calibration of photoelectric quantum efficiency. *Physical Review Letters* **117**, 110801 (2016).

[15] Hirano, T., Kotani, K., Ishibashi, T., Okude, S. & Kuwamoto, T. 3 db squeezing by single-pass parametric amplification in a periodically poled ktiopo4 crystal. *Opt. Lett.* **30**, 1722–1724 (2005).

[16] Kaiser, F., Fedrici, B., Zavatta, A., d'Auria, V. & Tanzilli, S. A fully guided-wave squeezing experiment for fiber quantum networks. *Optica* **3**, 362–365 (2016).

[17] Hajimiri, A., Abiri, B., Bohn, F., Gal-Katziri, M. & Manohara, M. H. Dynamic focusing of large arrays for wireless power transfer and beyond. *IEEE Journal of Solid-State Circuits* **56**, 2077–2101 (2020).

[18] Rogers, C. *et al.* A universal 3D imaging sensor on a silicon photonics platform. *Nature* **590**, 256–261 (2021).

[19] Valivarthi, R. *et al.* Measurement-device-independent quantum key distribution coexisting with classical communication. *Quantum Science and Technology* **4**, 045002 (2019).

[20] Benedikovic, D. *et al.* 25 Gbps low-voltage hetero-structured silicon-germanium waveguide pin photodetectors for monolithic on-chip nanophotonic architectures. *Photonics Research* **7**, 437–444 (2019).

[21] Notaros, J. *et al.* Ultra-efficient CMOS fiber-to-chip grating couplers. In *Optical Fiber Communication Conference*, M2I.5 (Optica Publishing Group, 2016).

[22] Michaels, A. & Yablonovitch, E. Inverse design of near unity efficiency perfectly vertical grating couplers. *Opt. Express* **26**, 4766–4779 (2018).

[23] Blumenthal, D. J., Heideman, R., Geuzebroek, D., Leinse, A. & Roeloffzen, C. Silicon nitride in silicon photonics. *Proceedings of the IEEE* **106**, 2209–2231 (2018).

[24] Leonhardt, U. & Paul, H. High-accuracy optical homodyne detection with low-efficiency detectors: "preamplification" from antisqueezing. *Phys. Rev. Lett.* **72**, 4086–4089 (1994).

[25] Nehra, R. *et al.* Few-cycle vacuum squeezing in nanophotonics. *Science* **377**, 1333–1337 (2022).

[26] Gurses, B. V., Fatemi, R., Khachaturian, A. & Hajimiri, A. Large-scale crosstalk-corrected thermo-optic phase shifter arrays in silicon photonics. *IEEE Journal of Selected Topics in Quantum Electronics* **28**, 1–9 (2022).

[27] Wang, K. *et al.* Quantum metasurface for multiphoton interference and state reconstruction. *Science* **361**, 1104–1108 (2018).

[28] Stokowski, H. S. *et al.* Integrated quantum optical phase sensor in thin film lithium niobate. *Nature Communications* **14**, 3355 (2023).

[29] Zhao, Y. *et al.* Near-degenerate quadrature-squeezed vacuum generation on a silicon-nitride chip. *Phys. Rev. Lett.* **124**, 193601 (2020).

[30] Dutt, A. *et al.* On-chip optical squeezing. *Phys. Rev. Appl.* **3**, 044005 (2015).

[31] Poulton, C. V. *et al.* Coherent lidar with an 8,192-element optical phased array and driving laser. *IEEE Journal of Selected Topics in Quantum Electronics* **28**, 1–8 (2022).

[32] Lim, A. E.-J. *et al.* Review of silicon photonics foundry efforts. *IEEE Journal of Selected Topics in Quantum Electronics* **20**, 405–416 (2014).

[33] Michel, J., Liu, J. & Kimerling, L. C. High-performance Ge-on-Si photodetectors. *Nature Photonics* **4**, 527–534 (2010).

[34] Alexander, K. *et al.* A manufacturable platform for photonic quantum computing. *arXiv preprint arXiv:2404.17570* (2024).

[35] Beling, A., Xie, X. & Campbell, J. C. High-power, high-linearity photodiodes. *Optica* **3**, 328–338 (2016).

[36] Jiang, Z. *et al.* High-power Si-Ge photodiode assisted by doping regulation. *Opt. Express* **29**, 7389–7397 (2021).

[37] Kim, I. I., McArthur, B. & Korevaar, E. J. Comparison of laser beam propagation at 785 nm and 1550 nm

in fog and haze for optical wireless communications. In Korevaar, E. J. (ed.) *Optical Wireless Communications III*, vol. 4214, 26 – 37. International Society for Optics and Photonics (SPIE, 2001).

[38] Lawrie, B. J., Lett, P. D., Marino, A. M. & Pooser, R. C. Quantum sensing with squeezed light. *ACS Photonics* **6**, 1307–1318 (2019).

[39] Aasi, J. *et al.* Enhanced sensitivity of the ligo gravitational wave detector by using squeezed states of light. *Nature Photonics* **7**, 613–619 (2013).

[40] Casacio, C. A. *et al.* Quantum-enhanced nonlinear microscopy. *Nature* **594**, 201–206 (2021).

[41] Taylor, M. A. *et al.* Subdiffraction-limited quantum imaging within a living cell. *Phys. Rev. X* **4**, 011017 (2014).

[42] Iwasawa, K. *et al.* Quantum-limited mirror-motion estimation. *Phys. Rev. Lett.* **111**, 163602 (2013).

[43] Xu, C. *et al.* Sensing and tracking enhanced by quantum squeezing. *Photon. Res.* **7**, A14–A26 (2019).

[44] Auzinsh, M. *et al.* Can a quantum nondemolition measurement improve the sensitivity of an atomic magnetometer? *Phys. Rev. Lett.* **93**, 173002 (2004).

[45] Slepyan, G., Vlasenko, S., Mogilevtsev, D. & Boag, A. Quantum radars and lidars: Concepts, realizations, and perspectives. *IEEE Antennas and Propagation Magazine* **64**, 16–26 (2022).

[46] Spedalieri, G. & Pirandola, S. Optimal squeezing for quantum target detection. *Phys. Rev. Res.* **3**, L042039 (2021).

[47] Chen, Y.-A. *et al.* An integrated space-to-ground quantum communication network over 4,600 kilometres. *Nature* **589**, 214–219 (2021).

[48] Sidhu, J. S. *et al.* Advances in space quantum communications. *IET Quantum Communication* **2**, 182–217 (2021).

[49] Kržič, A. *et al.* Towards metropolitan free-space quantum networks. *npj Quantum Information* **9**, 95 (2023).

[50] Liao, S.-K. *et al.* Long-distance free-space quantum key distribution in daylight towards inter-satellite communication. *Nature Photonics* **11**, 509–513 (2017).

[51] Wei, S.-H. *et al.* Towards real-world quantum networks: A review. *Laser & Photonics Reviews* **16**, 2100219 (2022).

[52] Tyler, G. A. & Boyd, R. W. Influence of atmospheric turbulence on the propagation of quantum states of light carrying orbital angular momentum. *Opt. Lett.* **34**, 142–144 (2009).

[53] DiAdamo, S., Qi, B., Miller, G., Kompella, R. & Shabani, A. Packet switching in quantum networks: A path to the quantum internet. *Phys. Rev. Res.* **4**, 043064 (2022).

[54] Laudenbach, F. *et al.* Continuous-variable quantum key distribution with gaussian modulation—the theory of practical implementations. *Advanced Quantum Technologies* **1**, 1800011 (2018).

[55] Qi, B., Lougovski, P., Pooser, R., Grice, W. & Bobrek, M. Generating the local oscillator "locally" in continuous-variable quantum key distribution based on coherent detection. *Physical Review X* **5**, 041009 (2015).

[56] Grosshans, F. *et al.* Quantum key distribution using gaussian-modulated coherent states. *Nature* **421**, 238–241 (2003).

[57] Jouguet, P., Kunz-Jacques, S., Leverrier, A., Grangier, P. & Diamanti, E. Experimental demonstration of long-distance continuous-variable quantum key distribution. *Nature photonics* **7**, 378–381 (2013).

[58] Liao, S.-K. *et al.* Satellite-to-ground quantum key distribution. *Nature* **549**, 43–47 (2017).

[59] Liu, H.-Y. *et al.* Optical-relayed entanglement distribution using drones as mobile nodes. *Phys. Rev. Lett.* **126**, 020503 (2021).
